# Wildlife management and conservation in South Africa: informing legislative reform through expert consultation using the Policy Delphi methodology

**DOI:** 10.3389/fvets.2025.1549222

**Published:** 2025-06-30

**Authors:** Elena Mercugliano, Magdel Boshoff, Arianna Dissegna, Adriana F. Cerizza, Luca Laner, Avery E. Indovina, Pierfrancesco Biasetti, Riccardo Da Re, Giulia Mascarello, Barbara de Mori

**Affiliations:** ^1^Department of Comparative Biomedicine and Food Science, University of Padua, Padua, Italy; ^2^Ethics Laboratory for Veterinary Medicine, Conservation and Animal Welfare, University of Padua, Padua, Italy; ^3^Department of Forestry, Fisheries and the Environment, Pretoria, South Africa; ^4^Department of Reproduction Management, Leibniz Institute for Zoo and Wildlife Research, Berlin, Germany; ^5^ETIFOR, Padua, Italy; ^6^Communication Laboratory, Istituto Zooprofilattico Sperimentale delle Venezie, Padua, Italy

**Keywords:** wildlife conservation, Policy Delphi, wildlife management, expert consultation, participatory approach, human-wildlife conflict mitigation, human dimensions, South African wildlife legislation

## Abstract

South Africa’s wildlife sustainable management requires cohesive, evidence-based policy development that balances conservation goals with socio-economic needs. This study employed the Policy Delphi methodology, based on subsequent questionnaire rounds, to gather expert insights on critical priorities for wildlife-related policy, focusing on four species: lions (*Panthera leo*), elephants (*Loxodonta africana*), rhinos (*Diceros bicornis* and *Ceratotherium simum*), and leopards (*Panthera pardus pardus*). Experts were divided into panels based on species and areas of expertise: hunting, management, translocation, research, and animal welfare. Through three rounds, which took place from March to July 2024, the study sought to pinpoint South African policy issues needing amendment, addition, or removal, gathering expert opinions to achieve 70% consensus and suggestions for integrating these into policies. A total of 60 experts accepted to participate, 14 compiled all three Delphi questionnaires, while 40 of them contributed to at least one round. In Round 1, 34 experts suggested 523 pertinent issues meeting the study criteria: 260 amendments, 233 additions, and 30 removals. In Round 2, 28 participants considered 363 issues relevant, of which 254 obtained final agreement in Round 3 by 19 experts, divided into 19 thematic categories. Moreover, in Round 3, 617 suggestions for integration into policies were collected. Overall, the analysis underscores that the experts preferred modifying existing policies rather than removing measures, emphasizing the adequacy of the policies with adjustments. The final list of issues confirmed at the end of Round 3 and their categories represent experts’ priorities for the four focus species management reforms in South Africa. Moreover, the insights highlight gaps in South African wildlife legislation, including improved definitions, consideration of local communities, and addressing data deficiencies for evidence-based management and conservation. By identifying key areas for legislative improvement, this study provides a framework for actionable strategies to enhance wildlife policy in South Africa, following the broader aim of protecting wildlife, and with the potential of having an impact beyond national boundaries.

## 1 Introduction

Wildlife management, intended as “the application of ecological knowledge to populations [.] in a manner that strikes a balance between the need of those populations and the needs of the people” (1), has become increasingly important as global biodiversity continues to decline, highlighting the complex challenge of integrating scientific and professional knowledge with the dynamics of wildlife, ecosystems, and human interactions (2, 3). This is particularly true for countries that host endemic species and biodiversity hotspots in areas with rapid urbanization, such as South Africa (4, 5). In these contexts, wildlife management decisions should also consider the complexity of socio-economic and political aspects (3, 4), taking into account that human activities impacting biodiversity affect the socio-ecological landscape as well, particularly for Indigenous People and Local Communities (IPLCs) (4, 6). To address some of these issues, international agreements, such as the Convention on Biological Diversity (7) and CITES (8), have been established to promote biodiversity conservation through frameworks, protocols and strategic plans; among the latter, the Kunming-Montreal Global Biodiversity Framework (2022) (9) encompasses in its 2030 goals various aspects regarding wildlife management.

In South Africa, wildlife management encompasses a diverse array of approaches tailored to distinct goals such as conservation, education, and research (10). Wildlife is managed across varied contexts—national parks, sanctuaries, game reserves—differing in practices like translocation, hunting, and research. These approaches have far-reaching implications for animals, managers, landowners, local communities, and other stakeholders, often involving unevenly distributed costs and benefits (11–14). For example, wildlife management on private land influences both economic opportunities and local livelihoods (15, 16), while human-wildlife conflict can challenge community perceptions of management choices (17). South Africa’s unique reliance on private wildlife areas—key hosts of national biodiversity—highlights the complex intersection of conservation and privatization (18, 19).

This complexity is mirrored in South Africa’s legislative framework, which operates across provincial, national, and international levels. Key national regulations, such as the National Environmental Management: Biodiversity Act (NEMBA) (20) and its Threatened or Protected Species (TOPS) Regulations (21), govern species protection (22, 23). However, legislative provisions for iconic species like elephants, lions, rhinoceroses, and leopards remain inconsistent. For instance, elephants are protected by dedicated norms and standards under the 2004 Biodiversity Act [The National Norms and Standards for the Management of Elephants in South Africa (24)], while rhinoceros trophy hunting is regulated through the Norms and Standards for the Marking of Rhinoceros and Rhinoceros Horn and for Hunting Rhinoceros for Trophy Hunting Purposes (25). Leopards are covered under specific Trophy Hunting norms (26), but there is no equivalent for lions. This disparity highlights the need for a more cohesive regulatory framework (27–29).

Achieving legislative consistency demands evidence-based policymaking that integrates scientific knowledge with insights from professionals, local communities, and NGOs (3, 30) requires “usable knowledge” that is scientifically robust, context-specific, and inclusive of stakeholder perspectives (31, 32). Internationally, participatory approaches like the ethical matrix have been employed to organize ethical standings of stakeholders (33), while methodologies such as the Delphi method have proven effective in informing policy development across fields including animal welfare, public health, and wildlife management (34–41).

In alignment with South Africa’s emphasis on evidence-based policymaking (42), to provide a more cohesive regulatory framework for wildlife management and cover the necessity for knowledge integration in policies, this study aimed to provide evidence- and experience-based foundation to support wildlife policy reform in South Africa, collecting issues, recommendations and priorities for the management of the focus species. A Policy Delphi method was applied to gather this information, consulting experts in management, hunting, translocation, research, and welfare of elephants, lions, rhinoceroses, and leopards. Through three iterative rounds of questionnaires, the study aimed to: (a) identify legislative issues to modify, add, or remove; (b) assess expert agreement on their relevance; and (c) collect recommendations for future legislation. The study systematically explores expert perspectives on reforming South African wildlife legislation for these iconic species.

## 2 Materials and methods

### 2.1 Delphi technique

The Delphi technique was developed in the 1950s as a participatory approach that facilitates a structured group communication process. The objective of this methodology is to obtain the highest possible level of consensus through consecutive questionnaire rounds concerning an issue, action or decision reaching a shared output (43). The collected responses and opinions are usually shared with participants in an aggregated form to approach and create the following round. Despite general guidelines having been outlined (44), this is a flexible method that allows for variations (45). It is especially helpful for complex issues and when information is scarce, as it aims to gather new insights based on experience (43). The initial round is traditionally devoted to information retrieval, while the following rounds are usually focused on creating a controlled group feedback in which participants can consult other experts’ opinions, rate, and comment, allowing interchange and eventual change in opinion (44), trying to reach a consensus (46). In a Policy Delphi, the aim is to find alternative policy options, investigating not only common but also divergent opinions (41, 46, 47). In this study, the Policy Delphi was employed because of its characteristics (e.g., structured communication, flexibility, etc.), along and replying to the necessity to improve wildlife policies, as already successfully obtained for agrifood policies (41) and ecosystem-based management (48). In general, the Delphi technique proved to be effective also in identifying key animal welfare indicators (35, 49) and for ecosystem modeling and management based on knowledge (50). It was decided to have three Delphi Rounds with a consensus set at 70%, as in other studies, where it is generally set at 70–80% [i.e., (46)].

### 2.2 Ethical approval

The study was carried out in compliance with the relevant ethical and normative guidelines of South Africa [Protection of Personal Information Act No. 4 of 2013 (51)], Europe [EU Reg. 2016/679 (52)], and Italy. Participants voluntarily agreed to participate before the beginning of the study and they could withdraw at any time by contacting the team managing the study by email. At the beginning of each round, a privacy notice and written informed consent were provided to inform and assure anonymity and confidentiality. We informed the experts that by accepting the privacy and informed consent terms, they agreed to participate, with the understanding that their responses could be reported without being linked to their identity. Participants were notified that the information and data collected would be used only for research purposes and analyzed in an aggregated way. The study received also ethical clearance by the Ethics Committee of the Istituto Zooprofilattico Sperimentale delle Venezie (IZSVe) with the code Opinion CE IZSVe 18_2024.

### 2.3 Participants and recruitment

We defined “experts” by integrating an inclusive definition by Rowe and Wright (43) with the one given by Millar et al. (44): “Being ‘expert’ entails the acquisition of experience, special skills in, or knowledge of a particular subject and not necessarily the possession of academic qualifications.” Experts were recruited through a snowball sampling process (53) from 5 May 2023 to 13 March 2024, with a non-discriminative exponential approach, starting from an initial panel of 20 experts indicated by the Department of Forestry, Fisheries and the Environment (DFFE) of South Africa. Recruitment duration was linked to the necessity to meet the definition of “expert” and adequately represent the various categories. Each of these experts was individually asked to identify and suggest at least two other participants. In order to achieve adequate representation across the various panels, the minimum number of experts for each area of expertise was set at 10. The specific criteria for inclusion were: (a) being based in South Africa; (b) having at least 5 years of experience in the species/area they are nominated for; and (c) being outside the organization of the person who nominated them.

We invited experts to indicate their areas of experience (management, hunting, translocation, research, welfare) in terms of years for each species. Each expert could provide insights into more than one area or species if they fit the inclusion criteria. DFFE indicated three additional experts in animal welfare, hunting, management, and research fields, allowing us to increase panel diversity while maintaining the number of experts in each area, and ensuring panel balance. Experts were excluded if they did not reply to confirm their areas of expertise, if they were no longer based in South Africa, or did not meet the selection criteria. At the end of the recruitment, an email was sent to the participants to notify: the imminent beginning of the study, including a brief overview of the process structure and the Delphi methodology (44); that their responses would be considered for the following steps only if they were provided in their areas of expertise, but that all results would be presented to the DFFE; that only complete questionnaires (sent by clicking the submit button) would be analyzed for results. If experts did not complete or participate in one of the rounds, they could still take part in the following rounds. They were also informed about the practical use of the results for legislation reform and the intention to produce a scientific publication.

### 2.4 Delphi process

The responses were collected for the three Delphi rounds and a pilot trial through the online survey software LimeSurvey (54). South African experts were engaged in the pilot study, which aimed to collect some issues and evaluate the content and approach appropriateness in order to create the first round of the Delphi based on the obtained results. Once the pilot study confirmed the clarity and comprehensiveness of the questions, as well as their formulation to gather specific insights meeting the study’s objectives, the Delphi rounds started. Each round lasted approximately 3 weeks, and reminders were sent to enhance the response rate (55).

In each round, a questionnaire for each species (rhinos, elephants, lions, and leopards) was created and organized in five areas (management, hunting, translocation, research, and welfare). The category hunting encompasses the issues regarding the chase and harvesting of wild animals (56), while the translocation one contains issues related to the “intentional movement and release of a living organism” (57). With the research area, the aim was to gather insights on wildlife research as all those studies in which the populations are the final beneficiaries of the obtained results (58). The categories welfare and management were defined, respectively, as the ability of an individual animal to suitably react to adverse internal or external factors (59), and as a comprehensive methodology (60) encompassing various aspects of wildlife conservation. At the beginning of each round, we provided instructions on how to complete the questionnaire, the contact information of the lead research team, and a mandatory informed consent form. Emails, questionnaires, introductions, and instructions were provided in English. To track responses, a unique ID was automatically assigned by LimeSurvey to each participant, to which only the research team had access, ensuring participants’ anonymity from each other throughout the process. Before the beginning of the study, pertinent documents were shared with the experts allowing participants to familiarize themselves therewith: the relevant current legislation, documents, reports, a table summarizing the documents provided, a list of acronyms and definitions to refer to Table 1, and the timeline of the entire Delphi process. At the end of each round, the results were analyzed and included in a Round report, which was sent to the participants.

**Table 1:** Relevant documents and acronyms provided.

Acronym	Documents
NEMBA (20)	National Environmental Management: Biodiversity Act, 2004
TOPS Regulations (21)	Threatened or Protected Species Regulations, 2007
TOPS Species (61)	Threatened or Protected Species – species list, 2007
CITES (8)	Convention on International Trade in Endangered Species of wild fauna and flora regulations, 2010
Elephant N&S (24)	National Norms and Standards for the management of elephants in South Africa, 2008
Trophy Hunting Leopard N&S (26)	Norms and Standards for the trophy hunting of leopard in South Africa, 2023
Rhino horn N&S (25)	Norms and Standards for the marking of rhinoceros and rhinoceros horn, and for the hunting of rhinoceros for trophy hunting purposes, 2018

Invitation to Round 1 (R1) was sent to all the experts who confirmed their participation, asking them to indicate issues that in their opinion needed to be: (a) addressed differently in the current legislation; (b) addressed in the new legislation that are currently not included in existing one; (c) deleted from the current legislation. For (a) and (c) points, participants could indicate the specific provision they referred to in the current legislation. Some issues were provided as examples to clarify how respondents were required to provide their open answers and specific provisions. For each area, each expert could indicate 10 issues to be addressed differently, 10 to be modified, and 10 to be deleted in the current legislation, using a maximum of 300 characters, spaces included to prevent excessive interpretation variations (44). The questionnaire had a page for each area with a specific space for leaving a comment. R1 data collection took place from 19 March to 10 April 2024.

The outputs obtained in R1 were used to create the questionnaires for Round 2 (R2), in which experts had to indicate for each issue: (a) each issue’s relevance through a 5-level Likert scale (from 1—Not relevant to 5—Extremely relevant) (62); (b) the motivation for their choice. Participants could also leave a comment at the end of the questionnaire and indicate additional issues to consider in subsequent steps, with the related ranking. The R2 consensus threshold was considered to be reached if 70% of respondents had chosen from 3 to 5 on the Likert scale (Moderately relevant, Quite relevant, Extremely relevant). Concerning issues wording and R1 results reporting for R2, we applied the conservative principle, maintaining the results as raw as possible in the report and following rounds (55) to align with experts’ opinions. Some issues were rephrased to integrate legislation specifications or enhance linguistic syntax, ensuring clarity, and some specific provisions were modified when the reference was not correct or incomplete (e.g., indicating the correct paragraph or adding the number of pages). If the specific provision was not provided, NA (Not applicable) was indicated in the results and maintained in the following rounds. R2 remained active from 8 May to 2 June 2024. At the beginning of the round, the link to the R1 report was provided to allow participants to consult the complete results aggregated and anonymous (including excluded issues and comments).

**Figure 1:**
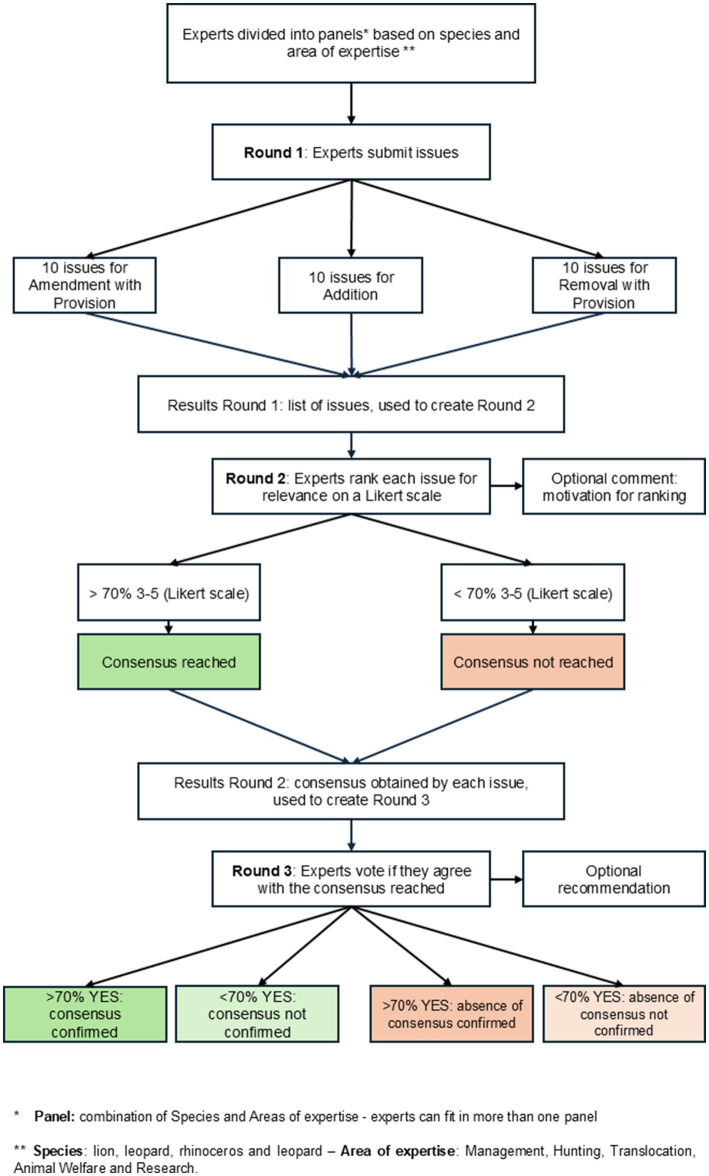
Flow diagram illustrating the study’s structure in the three rounds.

The results of R2 were presented to participants to be consulted and divided according to the consensus reached in the previous round, above or below 70%, and used to create the questionnaires for Round 3 (R3), active from 21 June to 14 July 2024. The standard deviation and the mean of the values obtained for each issue were included in the R2 report. In R3, issues were proposed to participants to collect (a) their suggestions on how to modify or introduce, in non-legal wording, each issue in the new legislation (recommendations); and (b) their agreement with the percentage of consensus reached in R2. We considered an issue confirmed by the panel if at least 70% of participants agreed with the consensus of R2. For issues proposed in the comment section of R2, experts had to indicate the relevance, motivation, and recommendation on how to include them in the legislation. A flowchart illustrating how the rounds are interconnected is shown in Figure 1.

After the end of the third round, an anonymous survey was distributed online through Google Forms to collect experts’ feedback on the Delphi process (44), its comprehensiveness and usefulness for the study aim, along with some demographic and professional questions to search for significance with results. The list of questions is shown in Supplementary Material 1.

## 3 Results

### 3.1 Participants

During the recruitment process, 106 experts were contacted, specialized in the four target species (lions, leopards, elephants, and rhinos—black and white) as well as the five areas: management, hunting, translocation, research, and welfare. Of these, 60 experts agreed to participate, meeting recruitment criteria and enhancing the diversity of expertise represented in the study. As the recruitment proceeded, we categorized the participants to ensure a broad and diverse group of specialists, obtaining an expectation of the number of replies in terms of compiled questionnaires for each panel and area (Supplementary Table 1).

Participants categorized according to different ranges of years of experience indicated by the experts are shown in Table 2, the majority having between 20 and 30 years of experience for all the species.

**Table 2:** Percentage of experts with different ranges of years of expertise for each species.

Species	From 5 to 10 years	From 10 to 20 years	From 20 to 30 years	From 30 to 40 years	Above 40 years
Elephant	11.50% (13)	29.2% (33)	45.13% (51)	11.50% (13)	2.65% (3)
Leopard	21.11% (19)	31.11% (28)	36.67% (33)	10.00% (9)	1.11% (1)
Lion	18.75% (21)	31.25% (35)	39.29% (44)	9.82% (11)	0.89% (1)
Rhino	14.03% (16)	21.93% (25)	55.26% (63)	7.02% (8)	1.75% (2)

Concerning participation in the three Delphi rounds (Table 3), the highest participation was in rhino hunting R1 (61.53%), while the lowest was in lion translocation R3 (9.52%). Experts who participated in at least one of the rounds were 40 of the 60 initially invited (66.67%). The total number of experts that participated in all the rounds was 14: nine in the elephant panel, seven in the leopard, eight in the lion, and six in the rhino. Of these 14 experts, five had between 10 and 20 years of experience in at least one area and species; eight between 20 and 30, and one between 30 and 40.

**Table 3:** Number of experts that participated in each round, panel, and area.

**Species**	**Area**															**N. of experts who took part in at least one round**	**N. of experts who took part in all the rounds**
	**Welfare**			**Research**			**Translocation**			**Hunting**			**Management**				
	**RI**	**RII**	**RIII**	**RI**	**RII**	**RIII**	**RI**	**RII**	**RIII**	**RI**	**RII**	**RIII**	**RI**	**RII**	**RIII**		
**Elephant**	10 52.63%	7 36.84%	6 31.58%	15 60.00%	13 53.85%	10 40.00%	9 40.91%	10 45.45%	8 36.36%	8 61.53%	7 53.85%	6 46.15%	18 52.94%	15 44.12%	617.65%	**22**	**9**
**Leopard**	3 21.43%	6 42.86%	3 21.43%	838.10%	10 47.62%	733.33%	320.00%	533.33%	213.33%	430.77%	430.77%	215.38%	12 44.44%	12 44.44%	725.93%	**18**	**7**
**Lion**	7 35.00%	8 40.00%	4 20.00%	10 41,67%	11 45.83%	625.00%	523.81%	733.33%	2 9.52%	850.00%	637.50%	212.50%	14 45.16%	13 41.94%	825.81%	**22**	**8**
**Rhino**	6 30.00%	7 35.00%	3 15.00%	836.36%	11 50.00%	522.73%	936.00%	11 44.00%	312.00%	436.36%	545.45%	327.27%	15 41.67%	16 44.44%	925.00%	**23**	**6**

Percentages were calculated on the total number of invited confirmed experts for each panel and area.

### 3.2 Round 1 results

Of the 60 experts invited to join R1, 34 replied by completing at least one panel and area in the questionnaires. The overall number of issues proposed in R1 was 820. Of these, 297 issues (49 for lion, 57 for leopard, 123 for elephant, and 68 for rhino panels) were excluded from the following rounds to maintain consistency with the Delphi methodology for one of the following reasons: not correctly categorized in the areas or already included in other categories by other experts; inconsistency with the topic, aim, or methodology of the present study; lack of comprehensiveness; inconsistency with the instructions provided.

Therefore, the total number of conforming issues in R1 resulted in 523: 198 for elephant (37.86%), 117 for lion (22.37%), 116 for rhino (22.18%), and 92 for leopard (17.59%). As shown in Table 4, the majority of issues were proposed in elephant management (149), followed by lion management (66) and rhino management (63). Overall, management was the area with the highest number of issues in all the animal panels, while the lowest was always hunting (except for rhino, which has hunting compared to translocation).

**Table 4:** Number and percentage of issues for each area proposed to be amended, added, or removed in the legislation.

Species and areas	Amendments	Additions	Removals	Total
Elephant				
Management	92 (61.74%)	44 (29.53%)	13 (8.73%)	149
Hunting	3 (42.86%)	4 (57.14%)	0	7
Translocation	8 (57.14%)	6 (42.86%)	0	14
Research	4 (36.36%)	7 (63.64%)	0	11
Welfare	7 (41.18%)	7 (41.18%)	3 (17.64%)	17
*Total*	114 (57.58%)	68 (34.34%)	16 (8.08%)	198
Leopard				
Management	22 (57.90%)	14 (36.84%)	2 (5.26%)	38
Hunting	4 (50.00%)	4 (50.00%)	0	8
Translocation	4 (36.36%)	7 (63.64%)	0	11
Research	9 (36.00%)	16 (64.00%)	0	25
Welfare	1 (10.00%)	8 (80.00%)	1 (10.00%)	10
*Total*	40 (43.48%)	49 (53.26%)	3 (3.26%)	92
Lion				
Management	37 (56.06%)	28 (42.42%)	1 (1.52%)	66
Hunting	4 (50.00%)	4 (50.00%)	0	8
Translocation	3 (33.33%)	6 (66.67%)	0	9
Research	5 (25.00%)	14 (70.00%)	1 (5.00%)	20
Welfare	8 (57.14%)	6 (42.86%)	0	14
*Total*	57 (48.72%)	58 (49.57%)	2 (1.71%)	117
Rhino				
Management	28 (44.44%)	29 (46.03%)	6 (9.53%)	63
Hunting	4 (66.67%)	2 (33.33%)	0	6
Translocation	2 (33.33%)	3 (50.00%)	1 (16.67%)	6
Research	4 (57.14%)	3 (42.86%)	0	7
Welfare	11 (32.35%)	21 (61.76%)	2 (6.06%)	34
*Total*	49 (42.24%)	58 (50.00%)	9 (7.76%)	116

Considering the modification of the legislation proposed, 260 issues were to be amended (49.71%), 233 to be added (44.55%), and 30 to be removed (5.74%). Overall, only in the elephant panel, the issues to be amended were the highest percentage, while in the other three panels, the issues to be added were the majority. In all the panels, the issues to be removed were the minority.

Table 5 shows some examples of issues proposed by experts to be amended, added to, or removed from the new legislation. See supporting information for the complete list of issues (Supplementary Data Sheet 1–4), including the excluded ones (Supplementary Material 2).

**Table 5:** Some examples of issues proposed by the experts.

Species	Modification	Issue of example
Management		
Elephant	Issue to be amended	Very important that pre-monitoring define exactly which elephant unit to be moved, to limit impact on remaining population
Leopard	Issue to be removed	Damage-causing animals (DCA) should not be used for commercial activities such as hunting. This will only lead to the “increase” of DCA leopards.
Hunting		
Rhino	Issue to be amended	Green darting \ hunting should be listed as restricted activity
Lion	Issue to be amended	Lions must be included under the list of Large Predators within the definition of TOPS, which would mean that provisions specific to large predators apply also to lions.
Translocation		
Elephant	Issue to be amended	Age limits and methods regarding translocation of calves require amending and specification.
Leopard	Issue to be amended	Translocation brings many risks to the animals involved and the people at the receiving environment, as well as costs. In the legislation it should be specified that translocation should be a last resort generally.
Research		
Rhino	Issue to be added	Non-invasive research (e.g., dung collection, observation) should not require veterinary ethics approval
Lion	Issue to be added	Trophy hunting and benefits to be studied.
Welfare		
Elephant	Issue to be added	Animals are collared too regularly due to collar failure, as better technology is not used due to costs. Animal welfare is therefore compromised in the process.
Leopard	Issue to be removed	There is little justification for the captive breeding of African leopards in South Africa from a conservation perspective, and captive breeding of leopards should thus not be permitted

**Figure 2:**
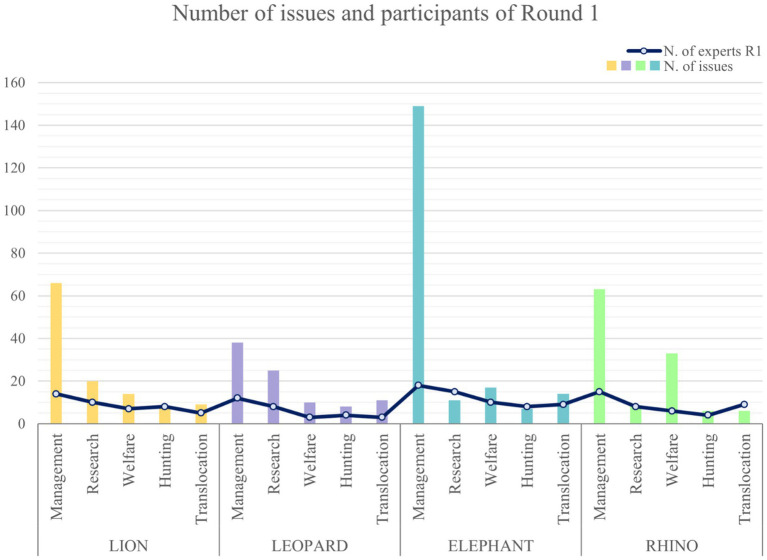
Number of issues and participants of R1 per panel and area.

Overall, Figure 2 shows the number of participants and issues proposed for R1 for each species and area. Even if the number of participants was lower than 20 in all the areas, the number of proposed issues differed among them, with higher experts’ contributions in management compared to the other areas and, apart from management, in rhino welfare and leopard research.

Regarding the documents cited by the experts in the issues suggested to be amended or removed from the current legislation, participants referred to documents (Table 6) not provided initially, which were collected and provided to the participants starting from the following round.

**Table 6:** Additional documents named by the experts during R1.

Acronym	Document
White paper (63)	White Paper on the Conservation and Sustainable Use of South Africa’s Biodiversity, 2022
Policy position (64)	Policy Position on the Conservation and Sustainable Use of Elephant, Lion, Leopard and Rhinoceros, 2024
PAPA (65)	Performing Animals Protection Act, 1935
APA (66)	Animals Protection Act, 1962
DCA N&S (67)	National Environmental Management: Biodiversity Act for damage causing animals, 2016
MTT report (68)	Ministerial task team report, 2024
HLP report (28)	The report of the High-Level Panel of experts for the review of policies, legislation and practices on matters of elephant, lion, leopard and rhinoceros management, breeding hunting, trade and handling, 2021
BMP lion (69)	Biodiversity Management Plan for the African lion (*Panthera leo*), 2015
NEMLA (70)	National Environmental Management Laws Amendment Act, No. 14 of 2013
TOPS TFS (71)	National Environmental Management: Biodiversity Act, 2004 (Act no. 10 of 2004)—Draft Regulations Pertaining to Threatened or Protected Terrestrial Species and Freshwater Species, 2024

**Figure 3:**
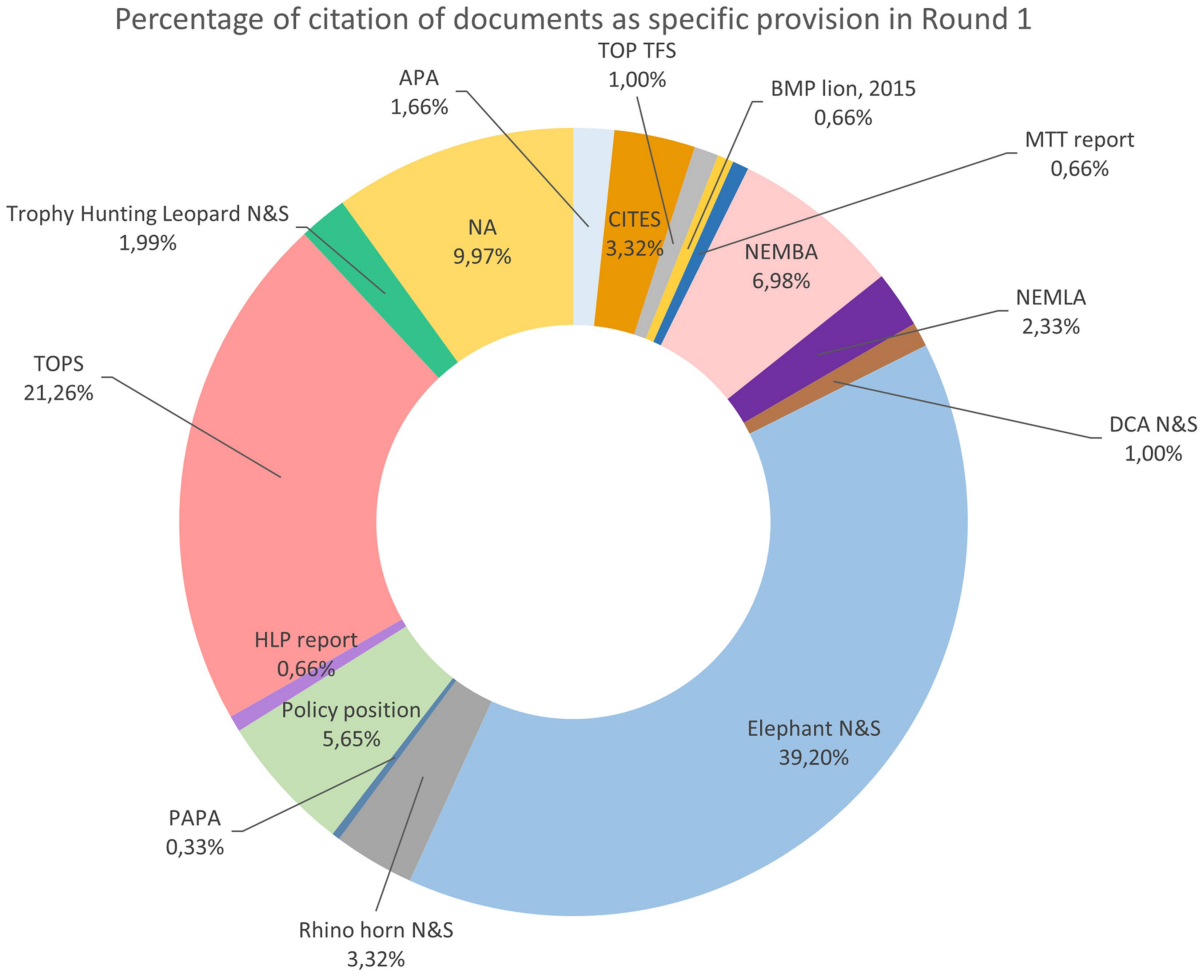
Percentage of citations for the documents provided by experts as specific provisions with the issues proposed in Round 1.

Overall, the experts cited all the documents a different number of times across R1, as shown in Figure 3 in terms of the percentage of the total number of citations. The three most cited documents were Elephant N&S (24) (39.20%), TOPS Species (61) (21.26%) and NEMBA (20) (6.98%).

### 3.3 Round 2 results

Of the 60 experts invited, 28 participated in R2 (46.67%). In R2, an increase in participation compared to the previous round was recorded for rhino, leopard, and lion panels, with the highest increase in leopard welfare and rhino research with three additional experts.

Of the 523 issues proposed in R1, 363 (69.41%) reached >70% of consensus: 117 in elephant, 93 in lion, 89 in rhino, and 64 in leopard panel (Table 7, consult Supplementary Data Sheet 5–8 for the complete results). The highest agreement was reached: for amendments in the welfare and translocation of elephant, leopard, and lion panels and research in rhinos; for additions in welfare and translocation in elephant and lion panels; translocation and research in rhino; and welfare in leopard panels (Table 7).

**Table 7:** Number of issues that reached 70% of consensus in R2 and related percentage on the total number of issues proposed in R1 as amendments, additions or removals for each panel and area.

Species and area	Amended	Added	Removed	Respondents
Elephant				
Management	50 (54.35%)	31 (70.45%)	5 (38.46%)	15
Hunting	1 (33.33%)	2 (50.00%)	-	7
Translocation	6 (75.00%)	4 (66.67%)	-	10
Research	2 (50.00%)	7 (100.00%)	-	13
Welfare	3 (42.86%)	4 (57.14%)	2 (66.67%)	7
Leopard				
Management	15 (68.18%)	11 (78.57%)	1 (50.00%)	12
Hunting	1 (25.00%)	2 (50.00%)	-	4
Translocation	4 (100.00%)	3 (42.86%)	-	5
Research	7 (77.78%)	11 (68.75%)	-	10
Welfare	1 (100.00%)	7 (87.50%)	1 (100.00%)	6
Lion				
Management	30 (81.08%)	20 (71.43%)	1 (100.00%)	13
Hunting	3 (75.00%)	3 (75.00%)	-	6
Translocation	3 (100.00%)	6 (100.00%)	-	7
Research	2 (40.00%)	10 (71.43%)	1 (100.00%)	11
Welfare	8 (100.00%)	6 (100.00%)	-	8
Rhino				
Management	26 (92.86%)	21 (72.41%)	3 (50.00%)	16
Hunting	1 (25.00%)	1 (50.00%)	-	5
Translocation	1 (50.00%)	3 (100.00%)	1 (100.00%)	11
Research	4 (100.00%)	3 (100.00%)	-	11
Welfare	8 (72.73%)	16 (76.19%)	1 (50.00%)	7

Two examples of issues that did not reach the consensus are: “Stockpiles of rhino horn in South Africa should be destroyed” 21.43% *(Rhino—management—added)*; “Identify properties with overabundance (for example, 200 elephants introduced in Madikwe Game Reserve—today they are more than 1,100) could be a huge threat to black rhinoceros population” 23.08% *(Elephant—management—added)*.

The mean relevance obtained considering all the issues was 3.47 for elephant, 3.75 for rhino, 3.91 for lion, and 3.63 for leopard. Only six issues reached the highest possible mean value of relevance in R2. Issues showing instead the strongest polarization were still six, with the highest standard deviation values and none of them reaching the 70% agreement (Supplementary Table 2).

One participant suggested an additional issue to be amended in the specific section of R2 for all the panels in the management area: “Must consider how the NEMBA Bill released for public comment on 24 May will impact this process.”

### 3.4 Round 3 results

Of the 60 experts invited, 19 (31.67%) filled in the R3 questionnaires: 13 in elephant, 10 in lion, 10 in leopard, and 9 in rhino panel.

R2 70% agreement was confirmed by experts for 254 issues: 81 issues in elephant, 59 in rhino, 70 in lion, and 44 in leopard panel (Table 8). The complete list of issues and results of R3 are shown in Supplementary Data Sheet 9–12. Of the confirmed issues, 123 were proposed as modifications, 134 as additions, and 4 as removals in the legislation. In total, 109 issues that reached 70% of the consensus in R2 were not confirmed by experts in R3: 36 for elephant, 30 for rhino, 23 for lion, and 20 for leopard.

**Table 8:** Number and percentage of the overall issues to be amended and added from R2 for which the consensus threshold > 70% was confirmed in R3, for each species and area.

Species	Area				
	Management	Hunting	Translocation	Research	Welfare
Elephant	59 (68.60%)	1 (33.33%)	7 (70.00%)	6 (66.67%)	8 (88.89%)
Leopard	17 (62.96%)	2 (66.67%)	7 (100.00%)	13 (72.22%)	5 (55.56%)
Lion	34 (66.67%)	6 (100.00%)	8 (88.89%)	9 (69.23%)	13 (92.86%)
Rhino	33 (66.00%)	2 (100.00%)	4 (80.00%)	6 (85.71%)	14 (56.00%)

Concerning not confirmed issues, experts did not agree with the percentage of consensus of 134 issues that in R2 did not reach the 70% threshold: 65 (80.25%) for elephants, 25 (92.59%) for rhinos, 24 (85.71%) for leopards and 20 (83.33%) for lions.

Concerning the additional issue proposed in R2 “Must consider how the NEMBA Bill released for public comment on 24 May will impact this process,” an agreement of 100% was reached, with 10 respondents, a mean of 4.6, and a standard deviation of 0.70.

In the survey area dedicated to inserting recommendations, experts proposed issues rewording, comments, or expressed their agreement with the issue or its original formulation. Experts provided 617 recommendations for the elephant, 344 for lion, 321 for rhino and 261 for leopard panel. Of these, the majority was regarding the issues that in R2 received 70% of consensus: 429 for elephant, 270 for lion, 258 for rhino, and 191 for leopard. For 33 issues, experts did not provide any suggestions.

Some comments agreed with the wording already proposed in R1. In other cases, as recommendations some experts changed the proposal from the R1 formulation. There were also issues in which the R1 content was considered valid but needed more specific wording, such as for the leopard translocation issue “Minimum monitoring should be implemented to evaluate the success of the translocation.” (R2 100%, R3 100%) commented “Definition of success will be important here, and is currently subjective.”

In R3 comments expressed concern about integrating some issues in the legislation: “This is good research, but cannot be legislated the way it is phrased [.].” In this case, the related issue was asking for scientific evidence: “Contraception has many negative side-effects. New research is needed into newer drugs with less negative side-effects.” (R2 consensus 85.71%, R3 60.00%.)

### 3.5 Results across rounds—overall perspective

Across the three rounds, experts did not compile a total of 17 surveys for leopard, 14 for rhino, 11 for lion, and 8 for elephant panels. Of the 14 experts who participated in all the rounds, 10 always compiled all the species related to their expertise.

**Figure 4:**
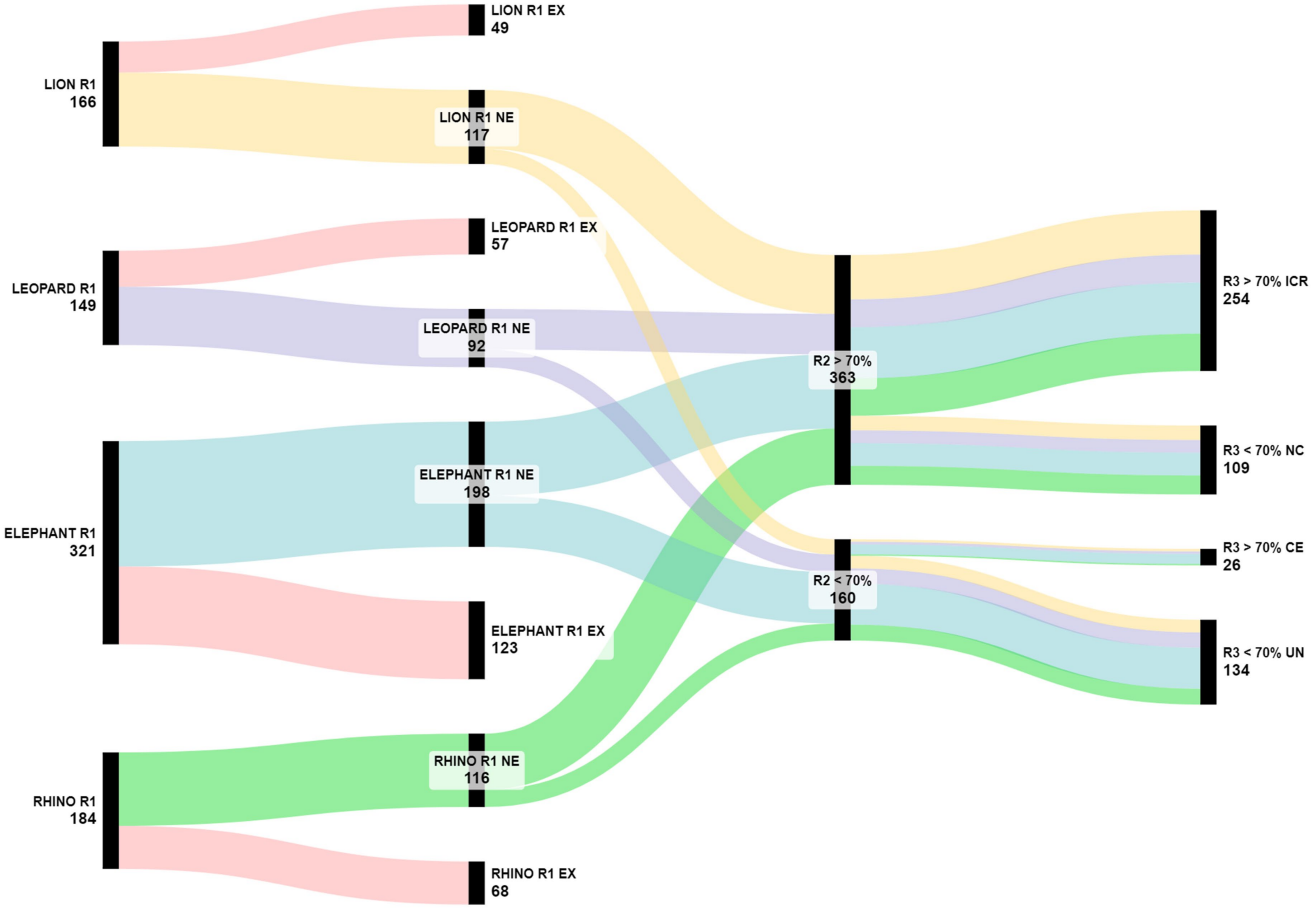
The flow of the issues across the three Delphi rounds starting from the total number of proposed issues, passing through the relevance indication by the experts, and landing on the agreement on the consensus reached in R3. EX, excluded issues (in red); NE, not excluded (lion in yellow, leopard in purple, elephant in blue, and rhino in green); >70%, issues that reached the consensus threshold; <70%, issues that did not reach the consensus threshold.

**Figure 5:**
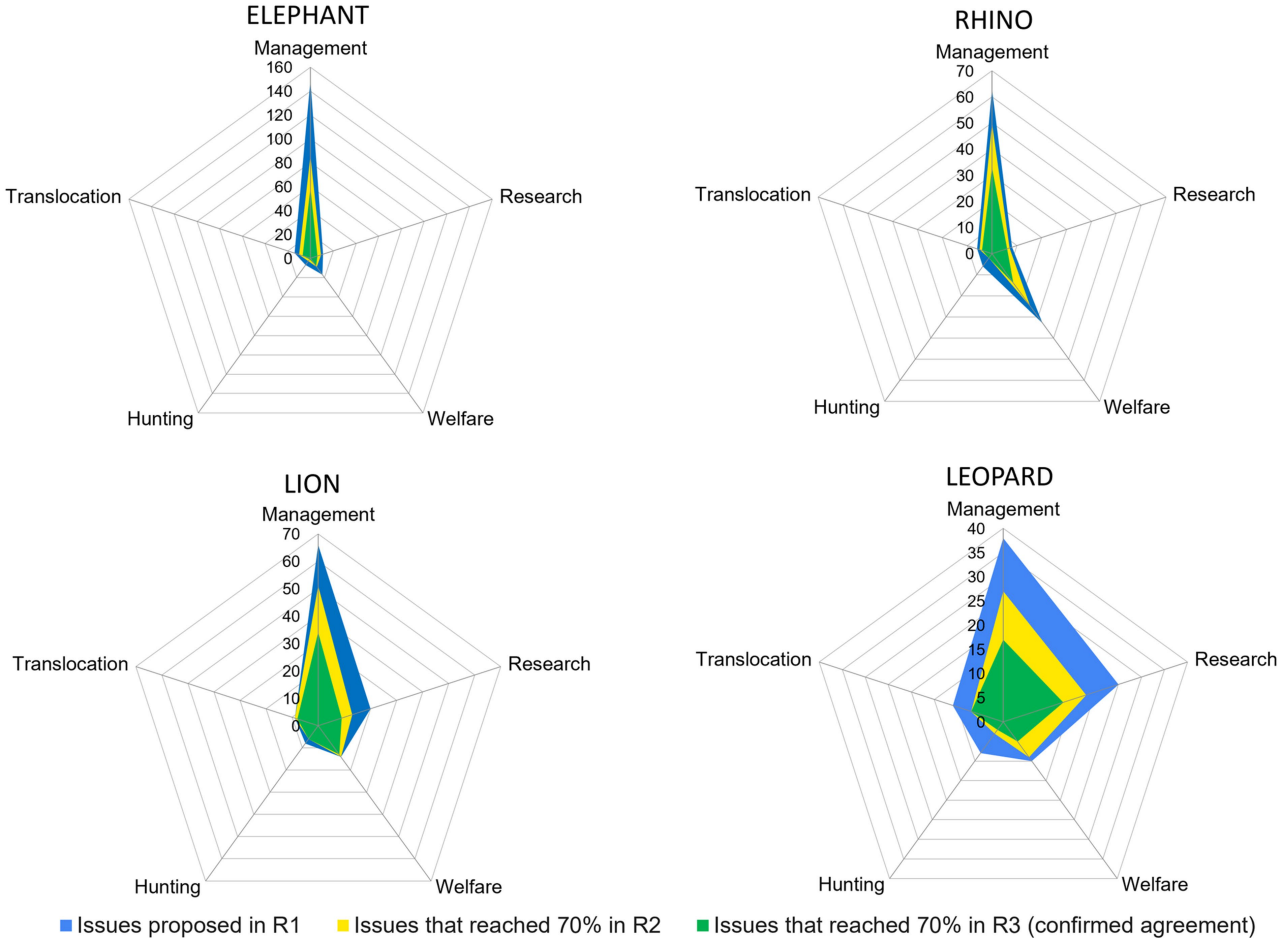
Number of issues proposed in R1, considered relevant in R2, and confirmed in R3 for each species and area.

Overall, R1 issues which kept 70% agreement across R2 till R3 were 254 (48.57% of the total; Figure 4): 81 (40.9%) for elephant, 59 (50.86%) for rhino, 70 (59.82%) for lion, and 44 (47.82%) for leopard (Figure 5).

### 3.6 Results categorization

**Figure 6:**
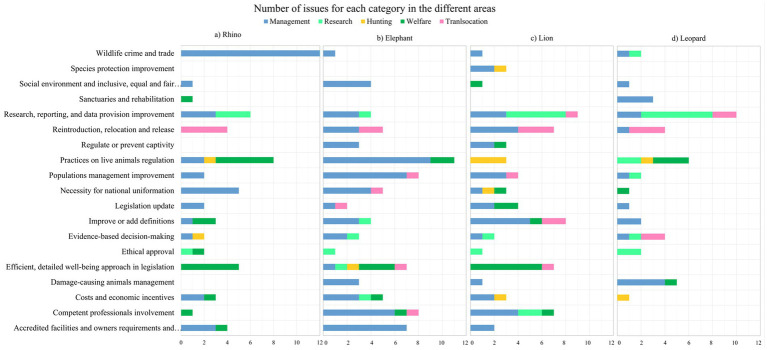
Number of issues in each category for the four panels.

The number of issues confirmed in R3 is shown in Figure 6, divided per topic into 19 thematic categories in the four panels. Definitions for each thematic category are provided in Supplementary Table 3, while the complete lists of issues with the associated category can be consulted in Supplementary Data Sheet 13. The partition into thematic categories revealed that the main topics that need attention according to the experts were: “Wildlife crime and trade” for rhinos; “Practices on live animals regulation” for elephants, rhinos, and leopards; “Efficient, detailed well-being approach in legislation” for elephants, rhinos and lions; “Research, reporting, and data provision improvement” for lions and leopards; “Competent professionals involvement” for elephants and lions.

In general, the application of an “Efficient, detailed well-being approach in legislation,” missing in the leopard panel, was required to be included in management, translocation, activities with animals, inspections and also penalties. In the category “Ethical approval,” experts’ issues required both to provide it for invasive procedures (*n* = 2), and to introduce exceptions to these documentations when dealing with non-invasive research and procedures (*n* = 4). Activities and procedures restriction (*n* = 16), regulation (*n* = 4), and permissiveness (*n* = 3) were required for “Practices on live animals regulation,” but also concerning other topics such as captivity, tracking, and telemetry collars, and the export of animals. Within the category “Costs and economic incentives,” 4 issues asked for incentives for owners (1 issue in the lion and 3 in the rhino panel). Two of the 16 issues within the category “Competent professionals involvement” required revising procedures under exclusive veterinarian responsibility to allow other professionals to carry them out. “Sanctuaries and rehabilitation” facilities were named only for rhinos and leopards. “Species protection improvement” was suggested only for lions in terms of considering the national conservation status of the species or listing it in the TOPS Large Predator list (61). “Wildlife crime and trade” control, management, and regulation included 3 issues regarding stockpile management (2 in rhino and 1 in lion panel); and other topics such as decreasing dehorning, illegal killing, and illegal trade. Only 2 issues accounted for respecting the traditions, inserted in the “Social environment and inclusive, equal and fair decision-making” category.

Focusing on issues that are not currently covered by the legislation proposed by experts to be added and confirmed in R3 (38 for elephant, 35 for rhino, 36 for lion, and 25 for leopard), they were mainly concerning “Research, reporting, and data provision improvement” (*n* = 17); “Efficient, detailed well-being approach in legislation” (*n* = 13); “Practices on live animals regulation” (*n* = 13); “Reintroduction, relocation and release” (*n* = 12), and “Wildlife crime and trade” (*n* = 11). No additions for elephant hunting were proposed and confirmed by the experts.

### 3.7 Feedback survey

**Figure 7:**
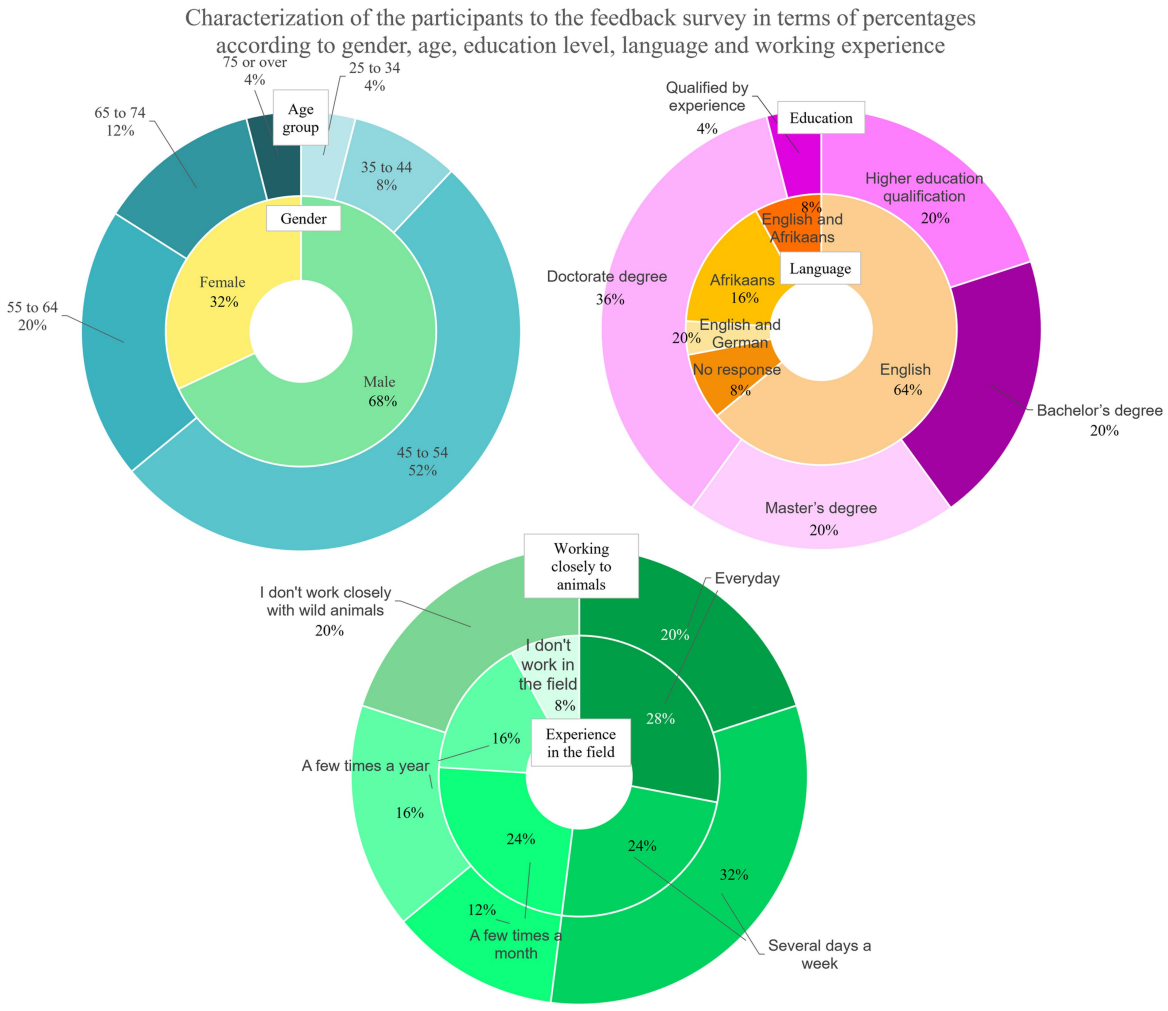
Characterization of the participants in the feedback survey according to gender, age group, education level, language, and working experience.

An overall perspective on the present study was extrapolated from the results of the non-compulsory feedback survey. In total, 25 experts filled in the survey, 8 (32%) females and 17 (68%) males. Figure 7 shows the characterization of the experts who filled in the feedback survey according to gender, age group, level of education, the main spoken language, and working experience in the field and close to animals (see Supplementary Data Sheet 14 for complete results).

**Figure 8:**
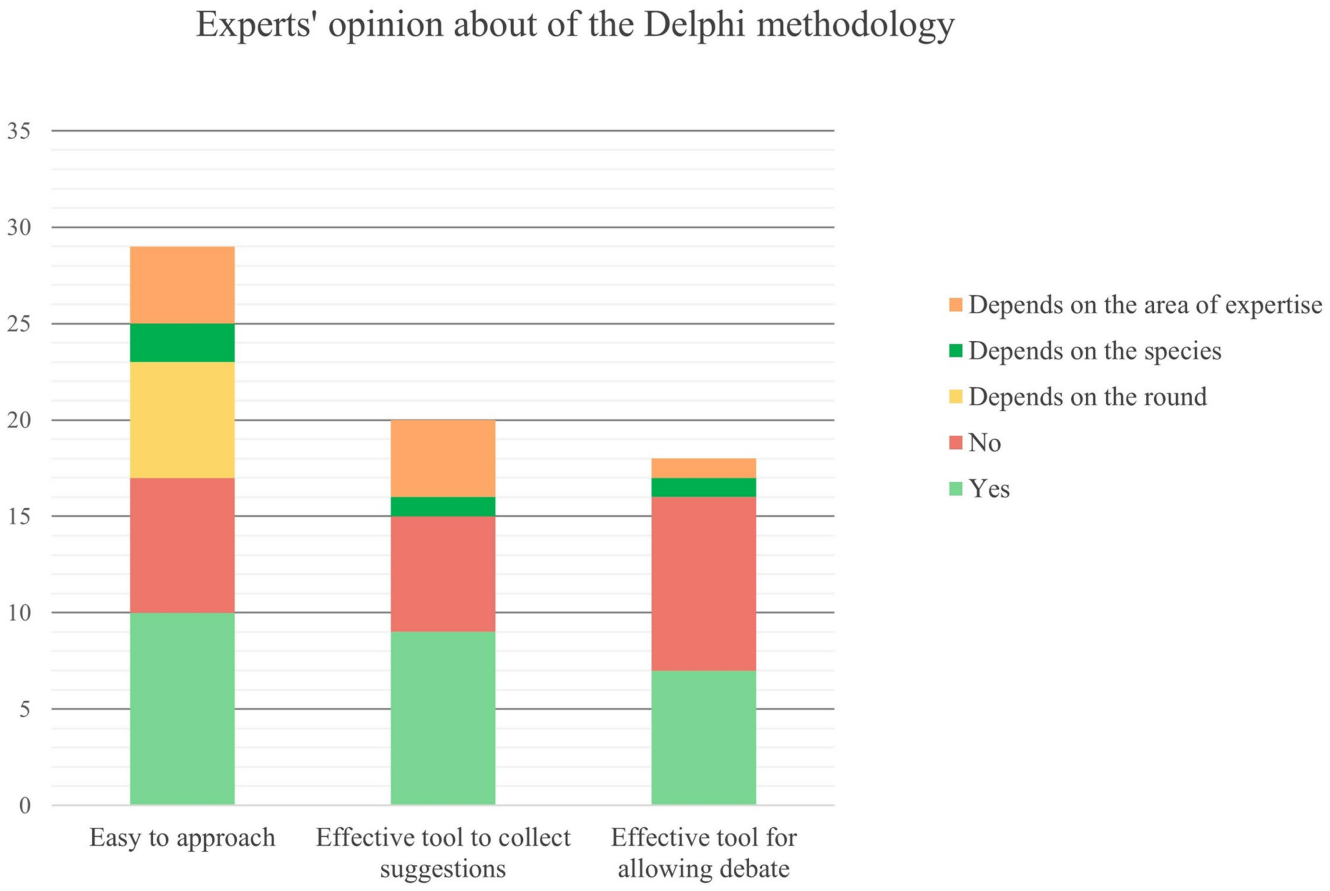
Experts’ opinions about the Delphi methodology: easy to approach, effective in collecting suggestions, and stimulating debate.

All the experts who filled in the feedback survey, except one, participated in at least one round (12–48%—in all the rounds and 10–40%—in two rounds). The following reasons were mainly indicated by experts for not having participated in one or more rounds: lack of time, too much time requested by the round, deadline missing, and technical or connection challenges. Despite these responses, 14 (56%) experts indicated that 3 weeks for the compilation was enough (3–12%—indicated that it depends on the round). Regarding the consultation of Round reports, 14 (56%) experts replied that they always consulted them, 5 not always, and one never. Five respondents indicated that they did not compile all the panels and seven all the areas of their competence for time constraints and topics overlapping, making “[.] it challenging to provide targeted answer.” Concerning recommendations in R3, 7 experts indicated they always gave them, 8 (32%) not always, 3 (12%) never, and 7 (28%) did not reply to the question. Motivations for not providing suggestions were: time constraints, repetitions, energy required, or they did not participate in R3. In general, experts indicated that the species, round, and area of expertise influence the Delphi method’s ease of approach, usefulness for collecting suggestions, and debate (Figure 8). Participants could indicate more than one answer to these questions.

## 4 Discussion

The present study aimed to fill the gap concerning knowledge provision for policy making and a more cohesive legislative framework while exploring the possibility of providing evidence- and experience-based support for policy reform, collecting experts’ insight concerning wildlife management. Through three Delphi rounds, this study collected issues to be modified, added, or removed in the South African legislation, investigating their relevance, and related recommendations on how to include them in future legislation. The results include a list of 254 issues confirmed as relevant by the experts for elephant, rhino, lion, and leopard management and divided into 19 thematic categories that represent priority reform topics.

The demand for evidence-based, informed legislation has grown to find alternative options to complex law issues and challenges (72). Even if there is a growing number of studies aiming to integrate science into policy, a few have been conducted to improve wildlife management legislation using the Policy Delphi method (73). As previous studies that aimed to provide a scientific basis for decision-making for nature management and conservation, our results identified key priorities (74) while showing wildlife management to be significantly influenced by specific regional and national aspects (75, 76). In addition, the identification of issues to be addressed in wildlife management have been proven effective through experts’ involvement in evaluating and improving policies, their comprehensiveness, coherence, and formulation (76). To our knowledge, this is the first study using the Delphi methodology to gather expert insights on South African wildlife management policies, innovatively engaging wildlife experts to develop shared, scientifically grounded recommendations for reforming legislation on iconic species management.

### 4.1 Participation across rounds

This methodology is based on panels’ experts and their participation across Delphi rounds (77). In the present study, we found different participants’ engagement across rounds, with a slight increase in R2 for rhino, leopard, and lion panels. This could be due to the willingness in R2 to know other participants’ opinions included in experts’ insights collected in R1 (44). For R3, lower participation could derive from the length of the process, exerting as a deterrent for participation (41) (R3 contained two separate questions, one about the consensus agreement reached in R2 and the other asking for recommendations).

Despite these discrepancies, 10 experts who participated in all the rounds always compiled all the species related to their expertise. In addition, 14 experts participated in all the rounds, strengthening and increasing, together with the high level of experience of the panel (the majority had between 20 and 30 years of experience in at least one area or panel), the study and results stability (77).

According to the feedback survey, one of the most cited motivations given by the experts for not participating or not filling in panels or areas of their competence was time constraints. Since Delphi studies typically are time-consuming and require a high effort, this aspect was explained to the panel at the beginning of the study to increase and ensure informed participation (44, 55). However, the higher effort invested in some specific areas and species could depend on the personal interest of the panel [as stated also by Truelove et al. (35)], motivation (44), the relevance of the policy issue under discussion (41), experience, and the current real or perceived necessities to legislate some aspects of species management.

### 4.2 Amendments, additions, or removals

In R1 the participation was higher in elephant management and hunting compared to other areas, probably because this is a charismatic species with a complex social structure (78). The highest number of issues proposed (92 to be amended and 44 to be added) in elephant management, compared to the other panels, could confirm the experts’ attention to the species and its management requirements. Other areas with a high number of issues were rhino welfare and leopard research. The first could be linked to dehorning, a recurrent topic across the rhino panel, and its welfare consequences (79). Concerning research aspects, it could be challenging to insert environmental evidence and science into legislation (30, 80). However, this could be even more difficult for leopards, for which South African research seems to fail to address specific conservation needs, potentially limiting research practice (81), and eliciting experts’ necessity to improve leopard research through legislation modification.

Overall, only in the elephant panel, the issues to be amended were higher than additions compared to the other areas. Since elephants have specific dedicated legislation regarding all management aspects (24), more amendments can be expected, revealing that the legislation should be modified according to experts. On the contrary, where the legislation is not present or exhaustive, experts could have suggested more additions, as they did for the other three species. The low number of additions proposed in panels and areas without specific legislation could be due to the low number of experts, their interests, and personal views about the completeness of the legislation for the current needs and activities. Overall, among the documents cited by the experts, the most mentioned legislation to be modified was the TOPS Regulations for all species (21), which could be considered a priority to be reformed by the government.

### 4.3 Consensus threshold

Concerning the agreement reached in R2, results could highlight a general accordance among experts on how to modify current guidelines and legislation. In some cases, such as for leopard research and rhino welfare, the high number of issues proposed in R1 underlined a great interest in the topic, but the low agreement suggests a potential polarization of opinions among experts. The amendment section of rhino and leopard hunting panels achieved the lowest agreement, with the latter possibly due to the new participants in R2 who could rank only issues proposed by others. For other panels and areas (such as elephant and lion research, and rhino hunting for amendments), comments for motivating ranking revealed that in some cases experts considered the issue relevant, but had different ideas on how to implement it into the legislation, explaining also contradictory comments compared to the ranking given by the expert. Two issues that received the highest agreement in R2 were about translocation and linked to respecting the rhino’s parental care needs and to assessing lions’ disease status before release, which has proven to impact translocation and release success (82). Even if for rhinos the translocation of mothers with dependent calves is not recommended (83), it was applied with success in the black rhinos (84). Black and white rhinos have a complex social organization (85, 86), a relevant factor for the success of both reproduction and relocation (84, 87).

### 4.4 Issues confirmation

At the end of R3, most issues were confirmed in their relevance agreement of R2. For elephant hunting, the area with the highest percentage of contacted experts participating in R1, the agreement obtained in R2 was confirmed in R3 only for 33.33% of the issues. In general, despite high levels of agreement obtained for lion and rhino hunting in R3, hunting received less attention from experts in R1 in terms of issues to be added to the new legislation for all the species. One of the reasons could be the recently recognized role of legal and regulated hunting for the sustainable conservation of South African species, as in the case of rhinos (88), together with the positive impact on the economy (89). In addition, even if the only South African legislation specifically dedicated to hunting is the draft Trophy Hunting Leopard N&S (26), specific provisions for elephants, rhinos, and lions hunting are already present and integrated into their dedicated legislation. Finally, the current international efforts to contrast, control, and punish illegal hunting (90) could confirm the completeness of measures, documents, and guidelines in place concerning this topic.

Another area with high agreement in R3 was leopard translocation, despite the lower participation in this area in R1 and the low agreement in R2. This could be linked to a recurrent topic in the issues confirmed in R3 for leopards: the management of damage-causing animals (DCA). According to McManus et al. (90), the majority of translocation events occurred because of human-carnivore conflict (HCC), one of the aspects that experts suggested to regulate in the legislation. Despite leopards can be successfully translocated under specific conditions (90), this practice can have negative impacts on conservation (91), potentially explaining the issues against translocating or exporting animals found in all the panels. On the contrary, for other species, such as the southern white rhinoceros (*Ceratotherium simum simum*), relocation contributed to preserving the species (92). Despite the positive impact of translocation, for many species, the entry into force of the Game Theft Act of South Africa in 1991 incentivized wildlife ownership together with the commercial translocation and export of animals and consequently their trade (92), removing the *res nullius* attribute of the species (93) as underlined by experts’ comments. Furthermore, experts’ concern about the link between translocation and commercial trade can be due to the lack of clear conservation purposes for translocation. For instance, in the Western Cape, the increase of translocated animals seems to be linked to the game ranching industry growth in the last decade (94), while the destination countries for the export of rhinos from South Africa between 2005 and 2016 included China (32.00%) (95). This could explain the high agreement reached also in R2 for the translocation area in all the panels.

Concerning the issues proposed to be removed, they were the minority already as proposals in R1, (*n* = 30), but only 4 were confirmed at the end of R3. This could highlight the necessity of modifying current documents and legislation instead of deleting measures already in place, which could have been considered correct according to the experts.

### 4.5 Issues with low agreement across rounds

The consensus of most of the issues that did not reach the threshold in R2 was not confirmed in R3. Even if experts could have changed between the two rounds, this consensus difference could derive from the issues’ complexity or wording. A deeper analysis through different research approaches of the topics included in these issues, as well as issues with polarized opinions, could provide insights into their inclusion in guidelines, acts, and legislation. For instance, focus groups, which are moderated discussions among individuals (96, 97) already used in wildlife research (98) and management (99), can be used to directly involve stakeholders in decision-making. Whereas decision trees can guide policy-making (80), providing a cause-effect model for wildlife management embracing the complexity of decisions and their consequences (100–102).

### 4.6 Thematic categories

The partition into thematic categories revealed that the most discussed topic was “Wildlife crime and trade” for rhinos, in which experts asked for transparency in these activities’ management, severe punishment for illegal hunting and trade, and investigation of illegal use of stockpiles by government officials. From 2012, the amended Rhino horn N&S (25) requires that all hunts, dehorning activities, and stockpiles are attended to and managed by officials (92). However, the corruption of certain NGOs, politicians, and government officials that seemed to follow the trade bans led to the consequent increase in illegal trade and poaching (92, 103), currently the main threat to the species’ survival (92). However, despite experts’ interest in this theme and the focus on stockpiles, the issues that proposed to destroy them did not reach the consensus threshold. On the contrary, all the issues that were confirmed in R3 for this topic asked to regulate the legal trade, in line with the recent scientific literature and reports [i.e., (87, 95, 104, 105)].

Experts also asked to regulate or ban “Practices on live animals regulation,” which issues referred to circuses, culling, trap use, and baiting. This topic, together with the experts’ request to respect or strengthen the requirements to own a wild animal, is linked to the responsible management of wildlife within facilities, particularly when they are conservation contributors (18). Responsible wildlife management is an interdisciplinary field in which the health, welfare, and biodiversity of individuals and populations, including genetics, are and should be considered both in the short and long term (18).

As part of wildlife responsible management, animal welfare is a transversal topic whose issues constitute the category “Efficient, detailed well-being approach in legislation.” Despite the lower participation in leopard welfare in R1, in R2 these areas for elephants, leopards, and lions obtained the highest agreement among experts, confirming the necessity to align the national legislative framework to the international one. Welfare concerns could also be the basis of the issues that requested to “Regulate or prevent captivity,” a category found only for elephants and lions. For elephants, this category underlined the attention to this species’ management needs and challenges in captive facilities (106). For lions, experts’ willingness to regulate captivity could be related to the debate about the breeding facilities of lions (29) in which two positions are found: one in favor because of captive breeding’s potential positive effect on wild populations conservation (107) and one against it for ethical and welfare concerns (108). These two positions have been found in our study’s comments for rhinos. These results are similar to what was found by Cousins et al. (109) for private lands captive breeding, in which stakeholders deemed wildlife ranches as conservation contributors only when they consider species’ threats and conservation recommendations. For some species, such as the southern white rhinos, captive breeding programs are (85) part of a conservation strategy, fostering population growth, preserving genetic diversity in the long term, but also animal welfare and safety (92). However, captive breeding programs’ success requires a cooperative management approach based on scientific information (92) and relevant and different stakeholders’ involvement (110). Among stakeholders frequently indicated by experts as needing to be involved, there are “Competent professionals,” despite in R2 one of the issues that received polarized ranking was about this subject. (“Regulations regarding staff experience for caring for captive lions should be established.”). Despite wildlife welfare being a recent field, as suggested by a participant, animal welfare specialists should “[.] be trained in wildlife welfare and not general animal welfare.” Indeed, specific wildlife management procedures can all compromise animal welfare at the individual and population level, making wildlife welfare a field of contact among management, animal welfare, and research (13). This interdisciplinarity was confirmed by those experts asking in the present study for ethical approval to be obtained for specific procedures.

### 4.7 Recurrent topics

Animal welfare and health were the focus of a recurrent topic concerning GnRH vaccine on elephants, for which use experts asked for restrictions “[.] in either male or female elephants until more scientific information is available.” This topic was also the focus of the only issue that received 5 as the mean value in R2. Even if the literature reports some benefits, there are also many uncertainties and scarce evidence about potential permanent health implications, negative and long-term effects, and their reversibility, which probably led experts to ask for restricted use (111).

Despite the existence of platforms such as Conservation Evidence (112), which can be used as a tool by policy makers and practitioners to make informed decisions, lack of data and evidence was another main issue indicated by experts, which, with data quality, and incorrect interpretation may influence conservation measures (113), such as translocation (90), management (81), welfare (108) and wildlife trade (113). In some cases, valuable records are lost due to red tape, as in the case of unclear procedures to request permits (114). Complex procedures and lack of national uniformity were underlined by experts in the different areas, for instance concerning activities and facilities permits, researchers’ access to samples, and a list of threatened species at a national level instead of criteria, categories, and definitions that currently vary among provinces (114).

The respect for indigenous traditions, linked with the use of materials, products, and derivatives, received experts’ attention despite opposing the necessity to take this aspect into account to the statement “Traditional and spiritual use should not be considered in conservation.” However, the traditional use and management of resources by Indigenous People and Local Communities (IPLCs) could be part of nature conservation and a matter of equity (4, 115).

A recurrent request made by experts was the improvement or addition of relevant definitions to the documents related to South African wildlife (17 issues were confirmed in R3 by experts), highlighted also by other works [e.g., (92)]. Despite some definitions containing relevant aspects according to experts, such as the balance between human and animals’ well-being (116), some participants stressed some imprecise definitions (e.g., sanctuary, metapopulation, wild managed, captivity, unnecessary suffering).

Finally, various themes and issues have emerged during the present study that require a deeper understanding, such as how much a private landowner should be responsible for their wildlife’s management, especially in economic and conservation issues. In fact, experts recurrently pointed out how necessary it is to incentivize owning a wild animal, with financial support, tax breaks, bureaucratic simplifications or changes in legislation from the government. Other issues, requiring deeper understanding, were about economic and social welfare linked to biodiversity conservation, adequate time frame and stakeholders inclusion for decision-making, precautionary principle to be applied in the legislation and ethical considerations. Together with the concept of transparency required by the experts, these aspects are related to conservation ethics in which four domains have been identified: social ethics, environmental ethics, animal welfare ethics, and research ethics (117). Future studies may focus on analyzing in detail these aspects to ensure fair and balanced policy-making with the inclusion of ethical principles in the legislation.

The Delphi method was an efficient approach to collecting wildlife experts’ insight about reforming South African legislation for the management of elephants, lions, rhinos, and leopards. The results of this study consist of a first step for the inclusion of evidence and experience-based knowledge into the South African legislation, building a list of issues and thematic categories that experts consider relevant to be addressed. This list could be used by policy-makers to improve wildlife management and set a baseline for evidence- and expertise-based national legislation with potential international resonance, supporting the efforts to protect elephants, rhinos, lions, and leopards.

### 4.8 Limitations of the study and future developments

Concerning recommendations, some experts did not interpret the request correctly, providing comments instead of formulating issues for their addition, modification, or removal from the legislation, posing a further challenge to qualitative data extrapolation and analysis. However, issues wording for professionals who are not policy-makers, even if in a non-legislative way, could be challenging, as highlighted by some experts in the feedback survey. Future studies could apply co-creation and participatory approaches, such as focus groups (96, 97), involving both experts and policymakers facilitating issues formulation for legislation reform, and ensuring a deeper participative analysis of issues that received a consensus slightly below the threshold, as well as those with polarized opinions.

The overlapping of expertise across panels and areas could be considered a study limitation since the research team had to compare competencies with responses received to exclude non-expert opinions. Indeed, participants’ expertise is crucial to collecting meaningful insights within a Delphi process since it is based and relies on expert involvement (44). On the other hand, distinct panels with dedicated experts could potentially enhance participation. However, some countries may not host a sufficient number of experts on a single species or area, making it necessary for the professionals to have a broader competence. Future studies could involve a higher number of experts in order to strategically employ participants’ expertise across panels. Regarding the areas of expertise, additional categories should be evaluated to be included in future similar studies (e.g., community engagement) in order to properly address all relevant competences.

Moreover, several international documents were included in the present study, thus, their reform should not be based exclusively on South African experts. Thus, including experts from other countries could prevent expertise overlap and increase panel numbers as well. The same Delphi methodology could be applied to other contexts and countries that host the same species. Indeed, apart from context-related aspects that can be managed at local level (e.g., Human-Wildlife Conflicts), the issues indicated by experts regard the focus species’ management from a general perspective (e.g., genetic and population management, reproduction, trophy hunting), potentially influencing the management approach to these species beyond South African boundaries. If these general needs emerge from a Delphi conducted in other contexts, these results could be intended as final categories and priorities to be implemented for conservation, providing also a supranational and international guideline for these species’ management.

## 5 Conclusion

This study aimed to provide evidence- and experience-based information for reforming South African policy regarding wildlife management while considering the necessity for a more homogeneous legislative framework. The inclusion of wildlife management aspects to generate evidence-based policies could be challenging. In particular, as highlighted also by experts during the present study, for specific aspects of wildlife management, such as research. Indeed, an issue could have been considered as a necessity to “[.] be addressed urgently” but without “[.] the need for it to appear in norms and standards as this could promote disinformation if not done correctly.” and “less of a priority [.] unless the effect is negative for the survival of individuals or the population.”

However, legislation reformation to include experts’ opinions and insight is even more important when regards threatened, charismatic, big-size species that are used and hosted in different contexts by diverse stakeholders. In addition, the national management of elephants, rhinos, lions, and leopards has an international impact because of their conservation status, tourism, and legal and illegal trade.

The present study aimed to explore experts’ insights for reforming South African legislation for the management of elephants, rhinos, lions, and leopards. The Delphi methodology proved to be a valid method to collect insights from different stakeholder groups, putting them in a dialog through the rounds through relevance ranking and comments. Through this approach, we were able to provide a list of issues to be amended, added to, or removed from the legislation considered relevant and confirmed by participants. Despite some of the issues proposed and the comments provided across rounds expressing opposite opinions about the same topic, the issues confirmed by experts in R3 underlined the necessity to regulate specific aspects. In addition, thematic categories identified the main topics gathered and that require amendment according to experts.

Despite the effort required to fill in the three rounds, the participation of the same experts in more than one round and the panel’s level of expertise ensured a stable process and reliable results. In addition, the results offered insight into conservation challenges in South Africa and in general for the subject species. The list of issues and thematic categories could provide support for the conservation of these species beyond policy reform. Wildlife managers, research institutions, and NGOs can be aware of the main challenges posed by the management of these species in the South African context, including legislation gaps and future potential reforms and requirements.

In conclusion, both for national and international scenarios that involve the four species included in the present research, this study represents the effort, willingness, and necessity to start a dialog between expertise and policies with the aim of improving species management, hunting, research, welfare, and translocation measures, with ultimate goal of their conservation in the present and their presence in the future.

## Data Availability

The original contributions presented in the study are included in the article/Supplementary material, further inquiries can be directed to the corresponding author.
