# Supplementary material for: Wildlife management and conservation in South Africa: informing legislative reform through expert consultation using the Policy Delphi methodology
Source: Front Vet Sci. 2025 Jun 30;12:1549222. doi: 10.3389/fvets.2025.1549222 (PMC12258392; doi:10.3389/fvets.2025.1549222)
Supplement: Supplementary file 1 [file Supplementary_file_1.docx]

**FEEDBACK SURVEY**

* Indicates compulsory questions

Informed consent

1. I confirm that I have read and understood the objectives of the present feedback questionnaire.*

(If you answer "yes" to this question, proceed, and submit the questionnaire, you are agreeing to participate in this survey. Answer to this question is mandatory to continue).

- Yes
- No

2. I confirm that I have read and understood the information on the processing of personal data.*

(If you answer "yes" to this question, proceed, and submit the questionnaire, you confirm to agree with the privacy policy and the processing of personal data.Answer to this question is mandatory to continue).

- Yes
- No

Feedback section

3. Please indicate your full name*:

_______________________________________________________________________

4. In which round did you take part during this study?

- Round 1
- Round 2
- Round 3
- None

5. If you did not participate in any of the rounds, please indicate why:

_______________________________________________________________________

6. If you participated in at least one round, do you think that the time given to reply - about three weeks - was adequate?

- Yes
- No
- Depends on the round
- Depends on the species
- Depends on the area of expertise

7. If you participated in Round 2 and/or Round 3, did you consult the reports provided with the results of previous rounds?

- Yes, always
- Not always
- Never
- I did not participate but I read the reports
- Other

8. At the beginning of the study, we asked you to confirm your species of expertise among elephant, lion, leopard and rhino.

Did you compiled the questionnaire regarding all the species you are expert in,within the round/s in which you participated?

- Yes, always
- Not always
- Never

9. If you have NOT always compiled the questionnaire regarding all the species you are expert in, please indicate why:

_______________________________________________________________________

10. At the beginning of the study, we asked you to confirm your areas of expertise among management, hunting, translocation, research, welfare.

Did you compiled the questionnaire regarding all the sections of the areas you are expert in, within the round/s in which you participated?

- Yes, always
- Not always
- Never

11. If you have NOT always compiled the questionnaire regarding all the sections of the areas you are expert in, please indicate why:

_______________________________________________________________________

12. During **Round 3** of this Delphi study, we asked you to give **recommendations** regarding issues in wildlife legislation to be amended, added or removed (Question: Please suggest how you would modify, in non-legal wording, each issue in the new legislation).

Did you compiled the questionnaire providing **recommendations**?

- Yes, always
- Not always
- Never

13. If you have NOT always compiled the questionnaire regarding recommendations, please indicate why:

_______________________________________________________________________

14. Did you find the Delphi method easy to approach and follow?

- Yes
- No
- Depends on the round
- Depends on the species
- Depends on the area of expertise

15. The purpose of this Delphi was to collect suggestions from experts for wildlife legislation in South Africa.

Do you think the Delphi method was an effective tool for the purpose of this work?

- Yes
- No
- Not sure
- Depends on the species
- Depends on the area of expertise

16. Do you think the Delphi method was an effective tool for allowing debate and communication among experts?

- Yes
- No
- Not sure
- Depends on the species
- Depends on the area of expertise

17. Do you think the overall effort required to experts by the process is worth the purpose of the project?

- Yes
- No
- Not sure
- Depends on the species
- Depends on the area of expertise

18. Do you think it was useful to include in the questionnaire a section about ethicalissues for the purpose of this project?

- Yes
- No
- Not sure
- Depends on the species
- Depends on the area of expertise

19. If you could choose one positive aspect of this project, what would it be?

_______________________________________________________________________

20. If you could choose one thing that should have been done better in this project,what would it be?

_______________________________________________________________________

Demographics

In this section, we will ask you for some **demographic information** to have **valuable insights** into our panelists and being able to perform **further statistical analysis**.

21. What is your age group?*

- 18 to 24
- 25 to 34
- 35 to 44
- 45 to 54
- 55 to 64
- 65 to 74
- 75 or over

22. What is your gender?*

_______________________________________________________________________

23. What’s your highest level of education?*

- No formal education
- Matric certificate
- Higher education qualification
- Bachelor’s degree
- Master’s degree
- Doctorate degree
- Prefer not to answer
- Other

24. What is your work status?*

- Employed in the public sector
- Employed in the private sector
- Self-employed/Freelance
- Manager in the public sector
- Manager in the private sector
- Work for a Non-Government Organisation
- Campaigning
- Homemaker
- Part-time
- Unemployed – Looking for work
- Unemployed – Not looking for work
- Studying
- Military/Forces
- Retired
- Not able to work
- Prefer not to answer
- Other

25. What language do you mainly speak at home?

_______________________________________________________________________

26. How frequently do you work in the field?

- Everyday
- Several days a week
- A few times a month
- A few times a year
- I don't work in the field

27. How frequently do you work closely with wild animals (including zoos and other type of facilities)?

- Everyday
- Several days a week
- A few times a month
- A few times a year
- I don't work closely with wild animals

Results

28. Do you want to receive information about the results of this study? If yes, please indicate your email address:

_______________________________________________________________________

Final comment

29. If you want, you can leave a final comment on this Delphi study:

_______________________________________________________________________
