# Supplementary material for: Wildlife management and conservation in South Africa: informing legislative reform through expert consultation using the Policy Delphi methodology
Source: Front Vet Sci. 2025 Jun 30;12:1549222. doi: 10.3389/fvets.2025.1549222 (PMC12258392; doi:10.3389/fvets.2025.1549222)
Supplement: Supplementary file 2 [file Supplementary_file_2.docx]

Round 1: Excluded Issues

For the study objectives and instructions/rules, for coherence and consistency, the following issues proposed in Round 1 have been excluded from the following rounds for one of the following reasons: inconsistency with the topic, aim, or methodology of the present study; lack of comprehensiveness; inconsistency with experts’ area of expertise; they were already reported in other parts of the round.

1. Lion
   1. Management

*Issues to be addressed*

| **Provision** | **Issue** |
| --- | --- |
| TOPS regulations | The prohibition of hunting captive bred lions should be lifted |
| NA | Streamlining permitting to facilitate management i.e. translocation etc. |
| TOPS regulations | Dogs must not be used to hunt lions even for problem animals |
| TOPS regulations | May need a pump action shotgun to kill a lion in self defence |
| TOPS regulations | TOPS - A section on how to implement the well-being of wildlife in management, which considers animals' sentience, their self-determination and agency, needs to be added |
| TOPS regulations | TOPS - A section on how to implement the well-being of wildlife in management, including the standards for transportation, needs to be added |
| TOPS regulations | Hunting a lion with a bow and arrow. Management: The anatomy of the lion and its posture (normally lying down) make it very difficult to kill it rapidly with a bow and arrow, raising serious ethical concerns. |
| NEMBA | Non-detriment Findings, the cornerstone of sustainable use and management, must always consider the well-being of animals, including at individual level |
| NEMBA | Non-detriment Findings, the cornerstone of sustainable use and management, must always consider the well-being of animals, including at individual level |

*Issues to be added*

| **N°** | **Issue** |
| --- | --- |
| 1 | DNA sampling of lions held in captivity |
| 2 | Compulsory for all captive held lion to be on PAAZA studbook |
| 3 | Stronger focus on metapopulation management principles |
| 4 | Accessibility of lion products and derivatives for traditional and spiritual use |
| 5 | Annual reporting on population estimates and management actions. |
| 6 | “Listed large predator” – will the lion not be added there at some point, or this even necessary to have still? |

- 1. Research

*Issues to be addressed*

| **Provision** | **Issue** |
| --- | --- |
| Policy position | Research pertaining to Policy Decisions and Legislation must be based on peer-reviewed publications. |
| TOPS regulations | Scientific research conducted in sanctuaries pseudo-sanctuaries and captive facilities - legitimacy and replicability. |

*Issues to be added*

| **N°** | **Issue** |
| --- | --- |
| 1 | Captive-bred lions may not be reintroduced into the wild but NBES strategy speaks to rewilding of captive lions based on one study with no peer-reviewed publications. |
| 2 | People always say something not researched, and research is needed, when in fact it has been done already |

- 1. Welfare

*Issues to be addressed*

| **Provision** | **Issue** |
| --- | --- |
| NA | Revise definition of wellbeing to duty of care concept |
| TOPS regulations | Must consult with experts |
| TOPS regulations | Must consult with stakeholders who have direct interest and/or are affected directly |
| TOPS regulations | Must consult with stakeholders who have direct interest and/or are affected directly |
| TOPS regulations | What is a "controlled environment" |
| TOPS regulations | Easily circumvented |
| TOPS regulations | Prevent hybridization/inbreeding. |

*Issues to be added*

| **N°** | **Issue** |
| --- | --- |
| 1 | No research without ethical clearance |
| 2 | Minimum enclosure sizes for all provinces regarding captive held lions |
| 3 | Norms and standards should be established for welfare of captive lions |
| 4 | Duty of care concept should be included |
| 5 | Norms and standards for husbandry of Lions in captivity should be established. |
| 6 | Animal-Visitor Interactions (AVI's) should be prohibited |
| 7 | Post mortems on dead lions conducted by a qualified veterinarian/pathologist should be mandatory with regular assessment. |
| 8 | Hunting quotas should be transparent and how they were derived should be opened to public debate/comment |
| 9 | Animals are currently classified as legal property or legal things in common law, making escaped lions fall to “res nullius principles”. This is very outdated based on rivers, animals etc been awarded non-human rights status in other parts of the world. Urgent review of status of animals is needed. |
| 10 | Heavily reliant on APA which is a criminal act and has its basis on "unnecessary suffering" - which is not defined. |

*Issues to be removed*

| **Provision** | **Issue** |
| --- | --- |
| NA | Definition of wellbeing |
| Policy position | The approval and continuation of breeding facilities for lions and other predators including exotic predators |

- 1. Hunting

*Issues to be addressed*

| **Provision** | **Issue** |
| --- | --- |
| NA | Baiting of wild lions should be permitted |
| NA | Incentivise the sustainable hunting of wild lion so that is it viable for land owners to own wild lion. Right now only the prime wildlife area can justify owning wild lion as the only viable revenue generator is photographic tourism in prime areas |
| NA | Offtakes should be the decision of the land owner |
| CITES | Increasing number of captive-sourced lions recorded as wild-sourced on CITES permits/database by provincial authorities. |
| TOPS regulations | TOPS does not allow put-and-take but provinces have different release periods. There is no uniformity across periods re-release periods prior to hunting. Still equates to put-and-take hunting as it simply allows for drugs to work out of animals system. |
| TOPS regulations | The hunting of DCA's should be removed. This is as controversial as is the categorization of a true "problem" or DCA. Animal should be destroyed as a management cull rather than a hunt from which commercial gain is achieved. Will prevent false categorization of DCA's as quick fixes. |

*Issues to be added*

| **N°** | **Issue** |
| --- | --- |
| 1 | Sustainable age limit placed on the hunting of wild male lion to ensure offtake is sustainable |
| 2 | The National Norms and Standards for Hunting Methods in South Africa have not been finalized to date - direct bearing on lion hunting. |
| 3 | Amended TOPS Regulations published for public comment in October 2023 need to urgently finalised as these have an influence on restricted activities and permitting |

*Issues to be removed*

| **Provision** | **Issue** |
| --- | --- |
| TOPS regulations  CITES | Trade in captive lion derivatives after captive lion hunting post the NSPCA judgement and no CITES quota |
| TOPS regulations  NEMLA | Use of poison to hunt damage causing animals including large-listed predators which include lions based on NEMLA Amendments |

*Comments*

| **N°** | **Comment** |
| --- | --- |
| 1 | With regards to all lion management practices and those that relate to wildlife utilisation and biodiversity conservation, there is an urgent need for the review and realignment of the regulatory and legislative framework and conflicting mandates between national and provincial management and permitting authorities and different departments i.e. Fishers, Forestry and Environment, Agriculture, Land Reform and Rural Development and Justice. The Ministerial Task Team Report has compiled a comprehensive Legislative Gap Analysis under 1.2.10 together with Table 6-1 Primary provincial nature conservation and biodiversity ordinances and policies, should be thoroughly reviewed and to demonstrate the lack of uniformity which better addresses the gaps than what the scope/character limits this questionnaire allows. |

- 1. Translocation

No issues have been excluded from Round 2.

1. Leopard
   1. Management

*Issues to be addressed*

| **Provision** | **Issue** |
| --- | --- |
| TOPS regulations | TOPS - A section on how to implement the well-being of wildlife in management, which considers animals' sentience, their self-determination and agency needs to be added |
| TOPS regulations | TOPS - A section on how to implement the well-being of wildlife in management, including the standards for transportation, needs to be added |
| Policy position | POLICY POSITION ON THE CONSERVATION AND S.U. OF LEOPARD - Regarding the hunting of leopard as Damage Causing Animal, consider the harvesting of DCA only when all mitigation measures have been effectively implemented and documented as failed |
| Policy position | POLICY POSITION ON THE CONSERVATION AND S.U. OF LEOPARD - Regarding the hunting of leopard as Damage Causing Animal, consider the harvesting of DCA only when a scientific and evidence-based system to correctly identify if the animal is truly a DCA has been put in place |
| TOPS regulations | Harsher penalties for non compliance - no longer able to take part in restricted activities |
| TOPS regulations | Snares are not legally defined, and there is no differentiation made between the use of foot loop traps or cable snares as a live capture method for research purposes, and lethal wire snares commonly used by poachers |
| TOPS regulations | There should be a consideration of whether a proposal has been assessed and approved by an animal ethics committee in the case of permits issued for research purposes |
| TOPS regulations | All options relate to removal of the damage causing animal (by culling or relocation), but there should be an assessment of whether reasonable steps have been taken to prevent damage taking place (e.g. is a farmer employing suitable livestock husbandry methods to reduce the risk of predation)? |
| TOPS regulations | Poison is an indiscriminate killing method and its use may result in the death of other individuals or different species scavenging on the carcass. |
| TOPS regulations | Recommend that bait (using the killed livestock) be added to this list |
| TOPS regulations | This also applies to animals originating outside a PA (*referring to "All options relate to removal of the damage causing animal (by culling or relocation), but there should be an assessment of whether reasonable steps have been taken to prevent damage taking place (e.g. is a farmer employing suitable livestock husbandry methods to reduce the risk of predation)?")*) |
| TOPS regulations | For all species not only leopard risk assessments should be done by specialists. Environmental assessment practitioners are not specialists.sonars |
| NA | Risk assessments should be carried out by registered professional scientist registered in terms of the Natural Scientific Professions Act |

- 1. Research

*Issues to be addressed*

| **Provision** | **Issue** |
| --- | --- |
| Leopard hunting N&S | Leopard N&S for hunting, Under 3. Add (14) The provinces may submit additional LHZs, provided comparable monitoring protocols as endorsed by the Scientific Authority who will then then adjudicate this. |

- 1. Welfare

*Issues to be added*

| **N°** | **Issue** |
| --- | --- |
| 1 | Penalties for illegal entrapment |
| 2 | The minimum standard for collaring leopard is with drop-off collars. This must be a permit condition with proof provided as part of the return process. |
| 3 | Methods to hunt DCAs must restrict methods - no dogs for example, in all Provinces. |
| 4 | DCA leopards which may be translocated must be fitted with a Satellite drop-off collar prior to release. |

- 1. Hunting

*Issues to be addressed*

| **Provision** | **Issue** |
| --- | --- |
| NA | There should be an obligation to consider other sources of offtake (damage-causing animals, removal through translocation, etc.) when issuing hunt permits for an area |
| NA | Hunting should only be permitted in areas where leopard populations are shown to be stable or increasing |
| N&S draft 2016 | The Scientific Authority should have the right to advise that hunting quotas should not be allocated to specific areas if scientific evidence demonstrates that the local leopard population is in decline or hunting could cause decline. This is alluded to but needs to be fortified and expanded. |
| N&S draft 2016 | In 2016 Draft Norms & Standards were produced for management & monitoring of hunting of leopard in SA |
| NA | These above mentioned N&S contained all the crucial issues to be dealt with through regulation. Not sure if final version was ever gazetted. |

*Issues to be added*

| **N°** | **Issue** |
| --- | --- |
| 1 | Provisions on access to leopard skins of DCAs for traditional and cultural use. |
| 2 | It might be advisable for trophy hunts to be required to supply a genetic sample from harvested leopards to the national biodiversity biobank South Africa. |
| 3 | If a female or an under-age male leopard is hunted, there should be a fine to the outfitter / client, along with the loss of a hunting quota the following season. |
| 4 | Hunting of Leopard should not be permitted |

*Comments*

| **N°** | **Comment** |
| --- | --- |
| 1 | The Leopard Hunting Norms and Standards provide a very solid basis for sustainable management of leopard hunting, and I would caution against any significant modifications to these regulations. The major concern is the lack of co-ordination between different forms of legal leopard offtake (hunting and damage-causing animal removal) - ideally if a LHZ has had a leopard removed as a damage-causing animal, there should be no hunting of leopards in that LHZ that year. It could be useful to contemplate mechanisms to allow for DCA leopards to be the subject of commercial hunts, although this does potentially pose some moral hazards |

- 1. Translocation

*Issues to be addressed*

| **Provision** | **Issue** |
| --- | --- |
| TOPS regulations | Provincial authorities transporting leopard |
| TOPS regulations | Haphazard trap setting for leopard |
| TOPS regulations | Cooperation on leopard with NGOs (interfering NGOs) |
| TOPS regulations | Release site choice of translocated leopard |
| TOPS regulations | Translocating leopard to extensive systems, or not |
| TOPS regulations | Translocation and release of rehabilitated leopard - sometimes rehab centres have warped ideas on release |
| TOPS regulations | Record keeping and research on translocations |
| NA | Rehabilitation facilities activities to do |
| CITES | Intra-African exports of leopard |
| CITES | Reinforcing other African countries with leopard |

*Issues to be added*

| **N°** | **Issue** |
| --- | --- |
| 1 | Translocations should take into account both the status of leopard populations in the source and receiving populations |
| 2 | Potential translocation introduction sites should be identified and subject to screening and risk assessment prior to any leopards being moved |
| 3 | Translocations should be done on the basis of benefit to the species or population, not individuals |
| 4 | A centralised database of leopard translocations should be developed and maintained |
| 5 | All translocations should take ecological, behavioural, genetic, disease and animal welfare considerations into account |
| 6 | Translocation introduction areas should be sufficiently large to support a viable leopard population |
| 7 | Socio-economic impacts of leopard translocations on communities around the release site should be considered |
| 8 | Impacts of the removal of individuals on the source population should be assessed and considered prior to translocation being approved |
| 9 | Translocation protocols |
| 10 | Translocation distances allowed for leopard |
| 11 | Where can leopard be translocated to and where not |
| 12 | Intra-African translocations when many invariably may die and there is a demand in Africa |
| 13 | What demographics is appropriate for translocations |
| 14 | Publications of results from translocations |
| 15 | Viable alternatives to translocations |
| 16 | Costs of translocations |
| 17 | When does lethal control trump translocation |
| 18 | Government agencies with a lot of translocation data not publishing |
| 19 | Translocations should be subject to post-release monitoring at the individual or population level |

*Comments*

| **N°** | **Comment** |
| --- | --- |
| 1 | None of the legislation provided contained much regulation of translocation, although this may be regulated through various provincial ordinances. Translocations of leopards do occur within South Africa, largely on a fairly ad-hoc basis, so greater regulation and the development of official guidelines/norms and standards, is needed |
| 2 | As with previous, it would have been labour intensive to seek which legislation to remove, and there is no abundance of it related these subjects, there are many sections I would delete, but they are not related to leopard translocation etc! An 'elephant in room' situation may present with this demand from other African countries to receive surplus, mostly problem leopard, which may invariably die if they remain here. They may be a loss to the country, but not to the continental population, its not as if they go into captivity and are lost forever. Genetically most of African leopard populations are connected anyhow, and it would be a way to enhance conservation benefit in other SADC countries. The NDF process is a workshop operating on what is available and one can be done within a single day, and should it target live exports for reintroductions elsewhere, I am pretty certain it will come up as of low detriment. Other than this, there needs to be more effort aimed at doing meta-analysis on the published information on leopard translocations and also to follow up on NGOs that are doing it all over the place without publishing, while government is also guilty of this - laptops have loads of collar data - but it never sees the light of day! |

1. Elephant
   1. Management

*Issues to be addressed*

| **Provision** | **Issue** |
| --- | --- |
| Resolution A10 | Trade should be allowed for commercial purposes of all aspects, currently trophies are for non-commercial purposes. Compliance is clearly the issue. |
| Resolution A10 | No large single sales should be permitted. this is not equitable and does not steadily feed the market over time which supports illegal trade. |
| Elephant management N&S | If no additional information is requested by the authority for a submitted management plan or there is no approval granted by the relevant authority for a submitted management plan within 60 days it should be assumed that the management plan is approved and can be implemented. |
| Elephant management N&S | Define "acceptable limits of change" in the context of Ch 2.1.8.2 |
| Elephant management N&S | Clarify who makes the decision of sedation as per Ch 2.12.1.c.4 since this is previously defined as a veterinary prerogative |
| Elephant management N&S | The preparation of PZP vaccines must be undertaken by a veterinarian but the administration must be done by a person with suitable marksmanship skill and experience which may or may not be a veterinarian |
| Elephant management N&S | Contraception can no longer be preferable to contraception. The latter has a worse effect on social and mental health of population |
| NA | NEMBA has been amended - S1 and S2. The current legislation - including N&S, Elephant Management Plans etc. to take cognisance of elephant sentience, wellbeing, welfare and protection at a species and individual level. |
| Elephant management N&S | Poor language - replace "this Act" with "the Act" |
| Elephant management N&S | Translocation restrictions currently in Elephant Norms and Standards |
| Elephant management N&S | Circus elephants must not be allowed into SA, and existing ones phased out |
| Elephant management N&S | Must make provision for extenuating circumstances |
| Elephant management N&S | Rehabilitation of captive elephants should be termed rewilding. |
| APA | The definition of well-being hinges on companion or domesticated animal criteria. Five domains in the South African legislation. This has no applicability to wild conditions. |
| Elephant management N&S | Vasectomies should be performed by specially trained vets. Elephant testes are located intra-abdominally and the laparoscopic surgery is highly technical and specialised. |
| Elephant management N&S | Add the placement of the shot should ensure immediate death, brain or heart shot |

*Issues to be added*

| **N°** | **Issue** |
| --- | --- |
| 1 | Support should be given for DNA testing of ivory to support claims of origin. |
| 2 | More regular sales compliant with CITES conditions should be allowed to avoid once-off flooding of the market which exacerbates demand and inequity of access to the product. |
| 3 | The establishment of new populations of wild elephants should also be made possible by allowing an existing population to access previously inaccessible areas (fence removal, green bridges/ tunnels, etc) |
| 4 | See: <https://emsfoundation.org.za/comments-on-the-draft-policy-position-of-the-conservation-and-sustainable-use-of-elephant-lion-leopard-and-rhinoceros/> |
| 5 | See: <https://emsfoundation.org.za/wp-content/uploads/2018/12/Comments-on-Elephant-Norms-and-Standards.pdf> |
| 6 | Trophy hunting of elephants must be prohibited. See: <https://emsfoundation.org.za/ems-foundation-commentary-on-the-proposed-2021-elephant-tusk-hunting-quota/> |
| 7 | <https://www.proelephantnetwork.org/2023/11/20/the-pren-submission-of-comments-on-the-draft-policy-position-for-sustainable-use-of-elephant-lion-leopard-and-rhinoceros-to-the-south-african-government-department-of-forestry-fisheries-and-the-env/> |
| 8 | Requirements for hunting of free-roaming elephants |
| 9 | Captive elephants that are translocated onto larger reserves for tourism, must only be moved together with their social bonding partners. Too often this does not happen creating huge welfare issues. |
| 10 | Importantly more consideration must be given to translocation of older bulls. Many people are moving bulls without considering the impact on the remaining population. e.g Addo hardly has any old bulls left! |
| 11 | Phasing out the utilisation of elephants at circuses |
| 12 | Elephant N&S - Ch 2 section 12 (6) |
| 13 | N&S - Chapt 2 - Part 5 - 25(2)(a) |
| 14 | Perhaps it is not new legislation needed, is consolidated legislation that is required. |
| 15 | Management of free-roaming elephants (res nullius) |

*Issues to be removed*

| **Provision** | **Issue** |
| --- | --- |
| Elephant management N&S | Delete "temporary holding facility" from the definition of "captive facility" since it is suitably defined under its own heading |
| Elephant management N&S | Delete "b) has escaped from an intensive wildlife system" since such an elephant has already been described as a roaming elephant |
| Elephant management N&S | Trophy hunting of elephants must be prohibited. See: https://emsfoundation.org.za/ems-foundation-commentary-on-the-proposed-2021-elephant-tusk-hunting-quota/ |
| Elephant management N&S | A plan of action needs to be put in place to phase out elephants in captivity in South Africa |
| NA | Translocation restrictions, elephants translocated before cant be translocated again |
| Elephant management N&S | The immunocontraception vaccine is not a scheduled drug/vaccine & does not need to be administered by a veterinarian as per current legislation. This vaccine needs be administered by a person with significant experience in elephant sexing, aging and behaviour for an effectively implemented program |
| NA | No breeding should be allowed in Captive facilities at all. |
| Elephant management N&S | Include elephant demographics |
| Elephant management N&S | Delete any reference to well-being as its not universally applicable to all situations in South Africa |

- 1. Research

*Issues to be addressed*

| **Provision** | **Issue** |
| --- | --- |
| Duty of care | If an elephant/s with collars too tight are encountered, the research project must be terminated. |
| Elephant management N&S | Annual feedback must be given, or research project relevance be reviewed by management authority. |
| Elephant management N&S | Accountability and responsibility from researchers |
| NA | Permit and research the testing of management programmes in regard to population reduction methods. Specifically wrt to simulating the effection predation by mad had on elephant and simulating the rest required for vegetation to rehabilitate |
| Elephant management N&S | There is no paragraph (3) under Research |
| Elephant management N&S | What is an appropriate data repository? |
| Elephant management N&S | Elephant research in wilderness areas should be wilderness dependent |
| Elephant management N&S | Immobilization and allowances for management methods not currently listed for "scientific research". Few management interventions that have not already been thoroughly contemplated. Animal Ethics Committee should also be independent of the institution proposing/ conducting the research. |

*Issues to be added*

| **N°** | **Issue** |
| --- | --- |
| 1 | Contravention of 28 .2 d (ii) puts a stop to the research project |
| 2 | Collared elephants continuously breaking out of, or causing damage are not exempt from being euthanized, and research program is accountable for damage. |
| 3 | N&S 2023 Part6 General /Research 28. Elephants should not be allowed to be darted for the sole purpose of research |
| 4 | Research can be done when an animal has to be darted for translocation or medical purpose |
| 5 | A minimum level of research and monitoring required for elephant management must be stipulated and detailed. |
| 6 | Long-term elephant research which informs legislation must be published by a certain time eg: to allow for registration of contraception drugs and avoid monopolies of access to such resources. |
| 7 | Parties/NGOs contesting legislation and treaties need to prove their case, it is not for Range States to defend their positions. All based on Science. |
| 8 | There is abundant research confirming that elephants are not suitable for captivity - see <https://elephantreport.net/wp-content/uploads/2022/07/Space-report-INTL2.pdf> |
| 9 | Captive elephants frequently suffer from foot and joint diseases, arthritis, weight related diseases, infertility, herpes virus, TB. See Miller et al 2016 Housing and demographic risk factors impacting foot and muscoskeletal health in elephants in zoos |
| 10 | Males require social networks comprising a wide range of potential social partners, particularly including older, prime, males. See Allen et al 2020 Importance of old bulls: leaders and followers in collective movements of all-male groups in African savannah elephants (Loxodonta africana) |
| 11 | Captivity damages the brain of elephants. See <https://www.researchgate.net/publication/354673378_Putative_neural_consequences_of_captivity_for_elephants_and_cetaceans> |

*Comments*

| **N°** | **Comment** |
| --- | --- |
| 1 | Failing to do to manage elephant populations is going to result in unsustainable impacts and local extinction of biodiversity, we absolutely need to understand this more. |

- 1. Welfare

*Issues to be addressed*

| **Provision** | **Issue** |
| --- | --- |
| Elephant management N&S | Elephant welfare is neither defined nor is it explicitly dealt with in the current Elephant N&S |
| Elephant management N&S | Contraception should not be promoted as more humane than culling - the body of evidence is not extensive enough. |
| Elephant management N&S | Translocation criteria are not specific enough to evaluate translocations and so there is lack of parity between Provinces and National eg: minimum property size. |
| Elephant management N&S | Intelligence of elephants is noted within the N&S but then say that they should not be accorded preference; this is conflicting - elephant self-reflexive consciousness should be acknowledged as special. |
| Elephant management N&S | Prohibition of keeping captive elephants in isolation |
| NA | Conflation of animal well-being and welfare with animal rights. |
| APA | The application of the five freedoms or five domains are not practical in wild conditions. Replace with a duty of car concept. |
| NEMLA | No reference to NEMLA Amendments re well-being whilst NEMBA is being revised. Should be included in Chapter 1 under "applicable legislation". NEMLA definition of well-being should be included in definition section. |
| Elephant management N&S | Animal Protection Act is from 1962 and Animal Welfare Law is currently being updated - this is a long slow process that cannot wait for the prescribed regulatory process. Needs interim action. Must rely on other law e.g. The UNEP resolution on animal welfare. |
| Elephant management N&S | Vets often used to make calls on welfare cases but they are not properly trained for full assessment. Animal's condition is based on more than just its physiological state. |

*Issues to be added*

| **N°** | **Issue** |
| --- | --- |
| 1 | No breeding should be allowed in Captive facilities at all. |
| 2 | Nowhere in the legislation is welfare mentioned as important |
| 3 | Keeping captive elephants goes against any welfare or wellbeing |
| 4 | When capturing and translocating elephants, welfare is rarely an issue |
| 5 | Disruption of social units goes against welfare principles and is not mentioned |
| 6 | High volume tourism goes against welfare and is not mentioned |
| 7 | Translocating of small groups goes against welfare and should be addressed |
| 8 | Historic ranges as well as duration of existence in those landscapes should be used to evaluate translocation to new areas. Areas may be in elephant ranges but they may have only been transient. |
| 9 | Adequate funding to enable monitoring and long-term care of the population needs to be stipulated prior to any introductions. |
| 10 | No reference to NEMLA Amendments re well-being whilst NEMBA is being revised. Should be included in Chapter 1 under "applicable legislation". NEMLA definition of well-being should be included in definition section. |
| 11 | The NEMLA amendments empower the Minister to prohibit certain activities that may negatively impact on the well-being of an animal. Therefore culling and hunting must be revisited. |

*Issues to be removed*

| **Provision** | **Issue** |
| --- | --- |
| Elephant management N&S | There is growing opposition to elephants in captivity based on ethical grounds citing welfare concerns. Tourists are also choosing destinations based on ethical choices. Thus captive facilities need to be urgently phased out. |
| Elephant management N&S | The NEMLA framework where animals well-being must be considered calls into question all lethal considerations including hunting and culling. This supports the removal of hunting of DCA's. |
| NEMBA | Reference to lions and other key species in White Paper and Policy Position as "faunal biological resources" should be removed. This equates animals to innate objects expressly for purposes of commodification only and doesn't consider their intrinsic value. |

*Comments*

| **N°** | **Comment** |
| --- | --- |
| 1 | See comments in the first section - management, hunting, research all need to take wellbeing, welfare and protection of elephants into account. All legislation pertaining to elephants needs to take their interests as a species and as individuals into account. |
| 2 | Only experienced vets and ground teams should do elephant work. If elephant need to be kept for some reason an independent elephant expert opinion should also be asked before just keeping elephants for certain reasons. |
| 3 | Get a rational group of people to develop rational welfare and ethical principles and keep the animal rights extremist groups far away from the process. They are presently the single biggest threat to wildlife conservation, simply because they devalue wildlife's ability to compete as a land use with other land uses. There must be an emphasis on true humaneness based on natural principles and not linked to industrial farming principles |
| 4 | See the comments with regards to the first question in this series. |

- 1. Hunting

*Issues to be addressed*

| **Provision** | **Issue** |
| --- | --- |
| NA | Needs to be less restrictive, and the decision needs to be put in the hand of the land owner, the same as is for other species such as wildebeest, zebra etc. You will then see the range of elephant expand and land owners will be able to justify owning elephant. Presently ownership is a burden. |
| Resolution A10 | Allow hunting for commercial purposes - this does not have to be trophy animals. |
| Elephant management N&S | Culling and hunting should also be tools for the spatial management of elephant |
| Elephant management N&S | Breeding cows where they occur without subadults can be hunted as a part of a total reduction program where the food can go to communities |
| Elephant management N&S | Hunting can be used to remove old bulls |
| Elephant management N&S | Updated information regarding the ecology and economics of trophy hunting elephant requires review/inclusion |
| Elephant management N&S | The hunting of escaped or roaming elephant requires independent assessment and verification |
| Constitution | Inclusion of duty of care principles in any form of sustainable use guidance in legislation. |
| Elephant management N&S | Hunting of a damage-causing animal. Should be a management cull. Int. or domestic hunter? Incentive to classify animals as DCA. |
| Elephant management N&S | Hunting of a damage-causing animal. Must be uniform across all provinces. Not all provinces are TOPS compliant so how is this managed? |
| Elephant management N&S | Hunting methods need to be assessed for DCA's. Inconsistency between N&S and TOPS. May not use an aircraft to hunt, may not drive an animal by any means, but an aircraft may be used for controlling a DCA? |
| NA | I have addressed this issue in the previous section. |

*Issues to be added*

| **N°** | **Provision** |
| --- | --- |
| 1 | Hunting of res nullius elephants |
| 2 | Allow the hunting of DCA animals under very strict conditions and monitoring in lieu of compensation payments. |
| 3 | Elephants must be added to the species list to which Regulation 24 and 26 (where applicable) of the Threatened or Protected Species Regulations (2007) apply |
| 4 | Bulls minimum two must be replaced every 8 years on small properties to prevent inbreeding. Old bull can become part of hunting |
| 5 | Hunting and culling should be revisited as the NEMLA amendments empower the Minister to prohibit certain activities that may negatively impact on the well-being of an animal. |
| 6 | Bulls of a specific age class and tusk weight agreed by elephant experts should only be considered to hunt. |
| 7 | Hunting of bulls is currently limited to solitary bulls not within the proximity of a social unit but this is not clearly defined. |
| 8 | Hunting of solitary males is not defined - are they of an age fully independent of their natal herd? How are the hunted bulls categorised according to the EMPs? |
| 9 | Measures of protection to protect big-tusked individuals need to be established to safe-guard large/potentially large tusked individuals from commercial exploitation and areas they are important for genetic variation and continuation and sexual reproduction and mate selection. |
| 10 | The National Norms and Standards for Hunting Methods in South Africa have not been finalized to date - direct bearing on elephant hunting. |

*Issues to be removed*

| **Provision** | **Issue** |
| --- | --- |
| Elephant management N&S | Delete "to the extent possible, should not be hunted if it is a collared elephant" |
| Elephant management N&S | Damage causing animals must be hunted within 24 hours after entering a property without a permit. The landowner must showed that he applied immediately after incident |
| TOPS regulations  Elephant management N&S | Hunting of a damage-causing animals should be removed. This is as controversial as is the categorization of a true "problem" or DCA. animal should be destroyed as a management cull rather than a hunt from which commercial gain is achieved. Will prevent false categorization of DCA's as quick fixes. |

*Comments*

| **N°** | **Comment** |
| --- | --- |
| 1 | These questions do not provide for strategic inputs and considerations. Hunting is one form of use and should be justifiable and sustainable. What is missing are guidance of responsibilities that people have towards a duty of care principle. A duty of care concept embraces reasonable measures to be taken to prevent harm from occurring to animals within the environment and biodiversity that they part off. It allows consideration and various options when harms that cannot reasonably be avoided or stopped, be minimized and rectified. An environmental duty of care thus inherently consider harm to animals and in the process embeds easily in thrivability concepts that enhance nature and people alike. The two are interdependent and human usage is central, including the use of animals. Because sustainability largely seeks to make sure things don't go worse, the concept of thrivability is far more useful especially when dealing with controversial uses like hunting. In this context somewhere legislation should embrace “Thrivability is the act of people and ecosystems thriving together though life giving rise to life that develop with vigour to prosper and flourish creating anti-fragile socio-ecological systems that enrich, generate, transform and strive for greatness of South Africa’s people and its natural heritage.” |
| 2 | There need to be clarity on CITES national quota vs provincial off-takes as well as to how the DCA's all feed into the national quota. This is important to understand how the different pieces of legislation apply to each other and ultimately to the numbers of elephant hunted. |

- 1. Translocation

*Issues to be addressed*

| **Provision** | **Issue** |
| --- | --- |
| Elephant management N&S | A wild elephant that has been previously translocated may not be translocated again. This is not practical and should be amended to "for management purposes" |
| NA | Restrictions on translocation - may not translocate previously translocated elephant |
| Elephant management N&S | In general release camps are no longer necessary. |
| Elephant management N&S | It is not necessary to sedate the elephants on release |
| Elephant management N&S | Circus elephants should not be allowed into SA and current ones phased out |
| NA | See comments and links in the previous section |

*Issues to be added*

| **N°** | **Issue** |
| --- | --- |
| 1 | I think that the elephant population outside Kruger should be managed as a meta-population through translocation. Maybe legislation should at least provide for this initiative without being prescriptive on how this should actually be done. |
| 2 | Translocation as a tool to mimic natural dispersal processes as part of elephant metapopulation management |
| 3 | It is very important that prior to capture pre-monitoring be done to ensure the entire and correct individuals are taken. |
| 4 | Capture operators take the first group they find hoping that all individuals are present, which mostly is not the case! This creates huge social disruption issues in remaining and new population |
| 5 | Elephants must be added to the species list to which Regulation 24 and 26 (where applicable) of the Threatened or Protected Species Regulations (2007) apply |
| 6 | Translocations driven by financial benefits for operators and funders rather than sound conservation or ecological benefits. |
| 7 | Translocated animals are not intact family groups many of the times! |

*Issues to be removed*

| **Provision** | **Issue** |
| --- | --- |
| Elephant management N&S | Prescription to make use of a release camp. Although a good practice it is not always applicable to every situation as free releases could be done successfully. |
| NA | Restrictions on translocation - may not translocate previously translocated elephant |

*Comments*

| **N°** | **Comment** |
| --- | --- |
| 1 | Only experienced veterinarians with elephant work done before along with other experienced veterinarians should be allowed to take on translocations. The translocation of elephants to happen safe and ethically should be done by an experienced team and not by any team and vet. |

1. Rhino
   1. Management

*Issues to be addressed*

| **Provision** | **Issue** |
| --- | --- |
| N&S for rhino horn | Local beneficiation through rhino horn |
| NA | The biggest issues that needs to be addressed is how to make the permitting and management of rhino more practical and enabling. It will also be much more efficient if rhino management is overseen from a policy and regulatory approach by the National Department of Environment only |
| CITES | Southern white or square lipped rhinoceros is currently listed as Appendix II CITES, the white rhino is threatened with extinction |
| Link 1 rhino | In 2023 499 rhinos were illegally killed, 406 rhino were killed on state owned properties |
| Policy position | There is no appetite to legalise the rhino horn trade why does South Africa pretend that there will be this opportunity? |
| CITES | South Africa is incapable of regulating or securing the rhino horn stockpiles which are supporting the illegal trade of rhino horn and fuelling the illegal killing of the remaining rhino |
| Link rhino 11 | Government officials are raiding the stockpiles of rhino horn |
| Policy position | The intensive breeding of rhino for horn for legal trade in South Africa was unsuccessful |

*Issues to be added*

| **N°** | **Issue** |
| --- | --- |
| 1 | If Norms and Standards are developed for TOPS registered facilities, and minimum standards or codes - then new legislation would not be necessary |
| 2 | Consideration of metapopulation management principles |
| 3 | Access and local beneficiation of rhino, rhino derivates and products |
| 4 | Meaningful participation in decision-making |
| 5 | Private rhino ownership that add to the conservation outcome of the national plan and are truly supporting rhino conservation should be explicitly recognised in the legislation |
| 6 | Timelines by when the respective government officials need to respond to comments and approvals need to explicit |
| 7 | In line with the above there needs to be a clear process that can be followed if government deadlines are not met and what the consequences to government officials not responding to their required responsibilities |
| 8 | Placing a limit to the number of rhino to a maximum of 20 that can be kept on a semi-wild facility. |
| 9 | As per section 1 |
| 10 | Issue 8 Contravenes 2.8 of the Biodiversity Management Plan of the white rhino |

*Comments*

| **N°** | **Comment** |
| --- | --- |
| 1 | The key issues with legislation is that it must enable conservationists. private sector, government implementing officials and supporting partners to work efficiently with each other. The current legislation is very disabling and almost a disincentive to have rhino on a property - be it state or private or community |

- 1. Research

*Issues to be addressed*

| **Provision** | **Issue** |
| --- | --- |
| NA | Rhino research should not be regulated by legislation but should rather enable management specific research outcomes that can inform better regulatory approaches |
| NA | Rhino horn stockpiles in South Africa are not regulated research has proved this. Horn from South African stockpiles is entering the illegal rhino horn trade. |

*Issues to be added*

| **N°** | **Issue** |
| --- | --- |
| 1 | Complete the South African Police Investigation that were initiated in 2015 in KwaZulu Natal |
| 2 | Complete the Investigation of the movement of arms from Mozambique into the Kruger National Park to KZN in 2016 |
| 3 | Research should be enabled and only be regulated by ensuring that any project that involves handling or restraining or immobilising a rhino is assessed by an ethics committee that is constituted by balanced and pragmatic approach |

*Comments*

| **N°** | **Comment** |
| --- | --- |
| 1 | Research is very important to rhino conservation and legislation needs to encourage proactive research that improves management decision making and population management of wild rhinos. Research that improves translocation and immobilisation approaches should also be encouraged. Pure academic research can be done opportunistically but should not be a priority over research that improves management and conservation of rhino |

- 1. Welfare

*Issues to be addressed*

| **Provision** | **Issue** |
| --- | --- |
| Link rhino 8 | Stop trophy hunting of rhino |
| NA | If dehorning is going to take place, all rhino must be dehorned in a short space of time |
| NA | No dehorning if environmental conditions are not suitable. Dehorning has become a tourism activity. Veterinarians are ignoring ethics on behest of such activities. |
| TOPS regulations | TOPS: The appointment of the Scientific Authority - the current list should include independent ethologists, zoologists, accredited welfare and rehabilitation specialists, and environmentalists. |
| Policy position | The documents lacks a section on welfare during transportation particularly national transportation |
| TOPS regulations | TOPS - A section on how to implement the well-being of wildlife, which considers animals' sentience, their self-determination and agency, needs to be added |
| TOPS regulations | TOPS - A section on how to implement the well-being of wildlife in management, including the standards for transportation, needs to be added |
| NEMBA | The Rhino Biodiversity Management Plan lacks the inclusion of animal well-being to be considered in all management procedures |
| NEMBA | The Rhino Biodiversity Management Plan lacks deep consideration of animal well-being when addressing the issue of dehorning |
| NEMBA | The Rhino Biodiversity Management Plan lacks considerations about rhino needs of socialisation, social structure and territorial behaviour |
| NEMBA | The Rhino Biodiversity Management Plan lacks considerations about rhino social needs, including suffering due to separation of family groups due to relocations and trade |

*Issues to be added*

| **N°** | **Issue** |
| --- | --- |
| 1 | Management of rhinos should be oriented towards wild, free roaming populations and not captive breeding. |
| 2 | The economics ecology, ethics and welfare considerations should result in the amendment of policy regarding the continuation of trophy hunting rhino. |
| 3 | On translocation the tips of all rhino should be removed to prevent damage during the process – this includes instances where the end destination prefer long horns. |
| 4 | Translocations should minimised and limited to strengthening existing populations (numerically or genetically), establishing new populations; maximising the growth rate of donor populations or rescuing threatened or orphaned individuals. |
| 5 | Rhinos should only be held in captive facilities for research that benefits rhinos, as a temporary measure within a translocation process or rehabilitating injured or orphaned rhino. |
| 6 | If dehorning must take place then the maximum removal must be encouraged rather than trimming to increase the length of interval between administration of anaesthesia |
| 7 | No dependent calf may be translocated without its mother and vice versa |

*Comments*

| **N°** | **Comment** |
| --- | --- |
| 1 | Rhino horn trimming should be standardised to prevent complications. As it was proven that certain trimming methods caused complications even when done by specialised vets. The normal square cut method is a good method if done correctly as it leaves a protective cover over the horn growth bed. If rhino horn was trimmed legally and ethically in the presence of a nature conservation official the owner of the horn should be allowed to donate the horn possible financial gain to look after the rhino welfare as the biggest constraint on rhino welfare is financial. |

- 1. Hunting

*Issues to be addressed*

| **Provision** | **Issue** |
| --- | --- |
| NA | Revision of guidelines in BMP on black rhino hunting tag allocation |
| CITES | The hunting of black and white rhino should be banned |
| Marking rhino horn N&S | The definition of "hunting client" must be amended to include anyone who is guided by a professional hunter in the hunting of a rhinoceros not just non-citizens and non-residents |
| NA | Hunting should be allowed if within an ethical and professional standard |
| NA | Hunting as per Appendix 2 currently should be continued |

*Issues to be added*

| **N°** | **Issue** |
| --- | --- |
| 1 | Annual reporting on population numbers and management actions / offtakes |
| 2 | The economics ecology, ethics and welfare considerations should result in the amendment of policy regarding the continuation of trophy hunting rhino. |
| 3 | The economics ecology, ethics and welfare considerations should result in the amendment of policy regarding the continuation of trophy hunting rhino. |
| 4 | The hunting of black and white rhino should be banned in South Africa. South African is incapable of protecting its rhino, they are not able to offer accurate figures of rhino in either the Kruger National Park or in Hluhluwe iMfolozi Game Reserve. |
| 5 | Rhino that are hunted must not be hunted in the presence of other rhino |
| 6 | Only lone bulls are to be hunted and not young bulls in natal crashes or older bulls associating with a herd. |
| 7 | Prioritization of Rhino related crimes with dedicated investigators |
| 8 | Same as above to make changes in include example in the current legislation. (referring to "*Only females older as 25 years may be hunted or proof from a veterinarian that they are not fertile. Add to animals that can be hunted.*") |

*Issues to be removed*

| **Provision** | **Issue** |
| --- | --- |
| Link rhino 9 | The hunting of black and white rhino should be banned |
| Marking rhino horns N&S | Remove "local hunter" from the definitions |

*Comments*

| **N°** | **Comment** |
| --- | --- |
| 1 | The hunting of rhino should be controlled to the sense that only animals older than a certain age to be hunted until numbers of the species is on the increase to a satisfactory number again. Once animal is legally hunted the owner should be allowed to charge an extra fee for the weight of the rhino horn to increase the value of the species and hope that this will initiate better private security if animals are more valuable alive for longer time periods. |

- 1. Translocation

*Issues to be addressed*

| **Provision** | **Issue** |
| --- | --- |
| Link rhino 10 | John Hume's experimental rhino breeding operation failed. 2000 rhino are going to be relocated across Africa. This is a necessary translocation. |
| NA | Only to places with adequate intelligence capability and proven counter poaching capability. |
| Policy position | No animals should be rewilded north of the Zambezi due to diseases that the animals are not resistant against. |
| Policy position | Only from wild populations to wild populations |
| NA | Rhinos need to be translocated by professional operators with the correct equipment and experience |
| NA | One national entity must be responsible for issuing the transport permit linked to the standing permits |

*Issues to be added*

| **N°** | **Issue** |
| --- | --- |
| 1 | No animals should be rewilded north of the Zambezi due to diseases that the animals are not resistant against - against the Biodiversity Convention. |
| 2 | On translocation the tips of all rhino should be removed to prevent damage during the process – this includes instances where the end destination prefer long horns. |
| 3 | Translocations should be minimised and limited to strengthening existing populations (numerically or genetically), establishing new populations, maximising the growth rate of donor populations or rescuing threatened/orphaned individuals. |
| 4 | Rhinos should only be held in captive facilities for research that benefits rhinos, as a temporary measure within a translocation process or rehabilitating injured or orphaned rhino. |
| 5 | One national entity needs to issue standing, transport and introduction permits so there is ability to have a central database, track effectively what happens with translocated rhino and adapt policy as lessons learnt. |
| 6 | Welfare standards need to be addressed as part of the legislation to ensure best practice is applied during translocations |

*Comments*

| **N°** | **Comment** |
| --- | --- |
| 1 | Translocations are an essential part of the both black and white rhino management. There exists tremendous expertise to do transactions correctly, these experienced operators should be enabled to operate effectively - so a standard registration that recognises good conduct and professionalism should allow these operators to operate in a more enabling environment. Permitting is mostly in place to regulate the permission to capture and translocate a rhino but does not indicate what are the welfare standards that need to be adhered to nor does it feedback into improving conservation outcomes for rhino. in the current format the permitting process does not materially contribute to the conservation of rhino but seems more like a barrier for efficiency and act as a cost centre for income to regulating authorities with no real benefit to the operators or rhino owners |
