# Supplementary material for: Wildlife management and conservation in South Africa: informing legislative reform through expert consultation using the Policy Delphi methodology
Source: Front Vet Sci. 2025 Jun 30;12:1549222. doi: 10.3389/fvets.2025.1549222 (PMC12258392; doi:10.3389/fvets.2025.1549222)
Supplement: Supplementary file 3 [file Table_1.docx]

**Number of expected questionnaire compilations for each panel and area, considering that some expert could take part in more than one panel and area.**

| **Species** | **Management** | **Hunting** | **Translocation** | **Research** | **Welfare** | **Total per species** |
| --- | --- | --- | --- | --- | --- | --- |
| **Elephant** | 34 | 13 | 22 | 25 | 19 | 113 |
| **Leopard** | 27 | 13 | 15 | 21 | 14 | 90 |
| **Lion** | 31 | 16 | 21 | 24 | 20 | 112 |
| **Rhino** | 36 | 11 | 25 | 22 | 20 | 114 |
|  |  |  |  |  | **Total compilations** | 429 |
