# Supplementary material for: Wildlife management and conservation in South Africa: informing legislative reform through expert consultation using the Policy Delphi methodology
Source: Front Vet Sci. 2025 Jun 30;12:1549222. doi: 10.3389/fvets.2025.1549222 (PMC12258392; doi:10.3389/fvets.2025.1549222)
Supplement: Supplementary file 4 [file Table_2.docx]

**Round 2: Issues with the highest Standard Deviation values across panels and areas.**

| **Species** | **Modification** | **Issue** | **St. Dev.** | **Percentage of agreement** | **Total N. of respondents/issue** |
| --- | --- | --- | --- | --- | --- |
| MANAGEMENT | | | | | |
| **Elephant** | Issue to be amended | Vasectomies performed by trained and experienced veterinary surgeons. | 2.07 | 66.67 | 6.00 |
| **Lion** | Issue to be added | Minimum safety requirements for staff and visitors at captive lions facilities should be established. | 2.00 | 55.56 | 9.00 |
|  | Issue to be added | Regulations regarding staff experience for caring for captive lions should be established. | 2.00 | 55.56 | 9.00 |
| HUNTING | | | | | |
| **Leopard** | Issue to be added | Hounds must not be used to hunt leopards. | 2.12 | 50.00 | 2.00 |
| RESEARCH | | | | | |
| **Lion** | Issue to be amended | Scientific authority members are not always qualified. On TOPS, reg 60, about Scientific Authority composition, add (4): The members contemplated in (1) b) to f) need to be suitably qualified at the minimum of a Masters degree in any of the biological sciences or nature conservation. | 2.00 | 62.50 | 8.00 |
| WELFARE | | | | | |
| **Rhino** | Issue to be amended | Training and capacitation of Environmental Management Inspectors (EMIs) to inspect and enforce the wellbeing mandate in NEMBA | 2.14 | 57.14 | 7.00 |
