# Supplementary material for: Wildlife management and conservation in South Africa: informing legislative reform through expert consultation using the Policy Delphi methodology
Source: Front Vet Sci. 2025 Jun 30;12:1549222. doi: 10.3389/fvets.2025.1549222 (PMC12258392; doi:10.3389/fvets.2025.1549222)
Supplement: Supplementary file 5 [file Table_3.docx]

**Table of definitions of thematic categories used to categorize issues confirmed in R3**

| **THEMATIC CATEGORY** | **DEFINITION** |
| --- | --- |
| Accredited facilities and owners requirements and responsibility | Issues related to permits, legal responsibilities, and criteria that must be followed by facilities and owners concerning animal management. |
| Competent professionals involvement | Related to the criteria and competencies required for professionals to operate and the kind of experts to be involved. |
| Costs and economic incentives | Concerning economic aspects and the need for incentives. |
| Damage-causing animals management | Issues related to the management of problem animals that cause damage to livestock, plantations or properties. |
| Efficient, detailed well-being approach in legislation | Concerning competent, detailed well-being approach inclusion in management, translocation, legislation, inspections, and penalties. |
| Ethical approval | It includes all the issues related to the need to introduce or exempt certain activities from ethical approval. |
| Evidence-based decision-making | Issues that stated the need for a scientific approach in decision-making and for scientific prioritization. |
| Improve or add definitions | Concerning the necessity of improving or adding definitions to the current legislation. |
| Legislation update | It includes the issues where a legislation update is proposed or updated guidelines are needed across all the themes (from staff competence to animal classification). |
| Necessity for national uniformity | It includes the issues stating the need for uniformity independently from the different provinces of South Africa. |
| Populations management improvement | It includes the issues suggesting improvement or modifications of management at the population and metapopulation level across all four species, such as management plans, translocation reports, and genetic management. |
| Practices on live animals regulation | It includes the proposed issues concerning live animals’ activities and the required criteria for their restriction or permission. |
| Regulate or prevent captivity | It covers all the issues in which experts asked to regulate or prevent animal captivity. |
| Reintroduction, relocation and release | Concerning a more precise and complete regulation of the different phases in reintroduction and relocation processes involving live animals to be released in the wild. |
| Research, reporting, and data provision improvement | The improvement of data collection, availability, transparency, and accessibility, involving all the stakeholders that are data providers and users, including researchers. |
| Sanctuaries and rehabilitation | Addressing permits, requirements, obligations, and information transparency of sanctuaries and rehabilitating centers and specific guidelines. |
| Social environment and inclusive, equal and fair decision-making | Issues asking for participatory approaches, relevant stakeholders representatives’ involvement, and different cultural needs equal consideration. |
| Species protection improvement | Concerning species conservation status and its relative protection. |
| Wildlife crime and trade | Related to illegal trade prevention, legal trade requirements, and the need to tackle wildlife crime. |
